# Variability in metabolic parameters and risk of dementia: a nationwide population-based study

**DOI:** 10.1186/s13195-018-0442-3

**Published:** 2018-10-27

**Authors:** Seung-Hwan Lee, Kyungdo Han, Hanna Cho, Yong-Moon Park, Hyuk-Sang Kwon, Gunseog Kang, Kun-Ho Yoon, Mee Kyoung Kim

**Affiliations:** 1 Division of Endocrinology and Metabolism, Department of Internal Medicine, Seoul St. Mary’s Hospital, College of Medicine, The Catholic University of Korea, Seoul, 06591 Korea; 2 Department of Medical Statistics, College of Medicine, The Catholic University of Korea, Seoul, 06591 Korea; 3 Department of Neurology, Gangnam Severance Hospital, Yonsei University College of Medicine, Seoul, 06273 Korea; 4 Epidemiology Branch, National Institute of Environmental Health Sciences, National Institutes of Health, Research Triangle Park, NC 27709 USA; 5 Division of Endocrinology and Metabolism, Department of Internal Medicine, Yeouido St. Mary’s Hospital, College of Medicine, The Catholic University of Korea, #10 63-ro, Yeongdeungpo-gu, Seoul, 07345 Korea; 6 Department of Statistics and Actuarial Science, Soongsil University, Seoul, 07040 Korea; 7 Department of Medical Informatics, College of Medicine, The Catholic University of Korea, Seoul, 06591 Korea

**Keywords:** Blood pressure, Body mass index, Cholesterol, Dementia, Glucose, Variability

## Abstract

**Background:**

Variability in biological parameters has been reported to be associated with adverse health outcomes. We aimed to investigate the composite effect of the visit-to-visit variability in blood pressure, glucose, cholesterol, and body mass index on the risk of dementia.

**Methods:**

A population-based cohort study including 2,930,816 subjects without a history of dementia, hypertension, diabetes mellitus, and dyslipidemia who underwent ≥ 3 health examinations was performed. The coefficient of variation (CV), standard deviation, and variability independent of the mean were calculated as variability indices. High variability was defined as having values in the highest quartile for each parameter.

**Results:**

A total of 32,901 (1.12%) participants developed dementia, of which 74.4% and 11.0% were attributable to Alzheimer’s disease and vascular dementia, respectively, during the median follow-up of 5.5 years. Individuals with higher variability of each parameter were at higher risk of future dementia. In the multivariable adjusted model, the hazard ratios and 95% confidence intervals of all-cause dementia were 1.22 (1.19–1.26) for one parameter, 1.39 (1.35–1.43) for two parameters, 1.54 (1.48–1.60) for three parameters, and 1.73 (1.60–1.88) for four parameters compared with subjects having no parameters of high variability measured as the CV. Consistent results were noted for Alzheimer’s disease and vascular dementia, using other indices of variability and in various sensitivity and subgroup analyses.

**Conclusions:**

There was a linear association between the number of high variability parameters and risk of dementia. Reducing variability of metabolic parameters would be a target to preserve cognitive reserve in the general population.

**Electronic supplementary material:**

The online version of this article (10.1186/s13195-018-0442-3) contains supplementary material, which is available to authorized users.

## Background

Dementia, a clinical syndrome affecting memory, thinking, and social abilities primarily caused by neurodegeneration, is becoming one of the greatest health and socioeconomic burdens in an aging society. The age-standardized prevalence of dementia for people aged 60 years or older was 5–7% in most world regions, affecting 35.6 million people in 2010 [1]. This number is expected to almost double every 20 years, with a higher rate of increase in low or middle-income countries [1, 2]. Although aging is the greatest but nonmodifiable risk factor, approximately 35% of the risk has been attributed to the combination of potentially modifiable risk factors including education, diet and lifestyle factors, psychiatric factors, and metabolic factors [2]. The presence of obesity, hypertension, or diabetes was associated with 50–60% higher risk of developing dementia, emphasizing the importance of managing metabolic and vascular risk factors [2].

Recently, visit-to-visit or day-to-day variability in biological parameters has emerged as a previously unrecognized residual risk factor, which is related to the development of various health outcomes. For example, higher blood pressure (BP) variability and lower heart rate variability have been linked to cardiovascular or cerebrovascular events and mortality [3, 4]. High variability in glucose or cholesterol levels was an independent predictor of mortality and vascular complications in subjects with diabetes or coronary artery disease, respectively [5, 6]. In addition, variability in body weight has been shown to have negative health consequences [7, 8]. These effects remained significant after adjusting for the mean levels of the parameters, suggesting that not only managing the absolute value but also reducing the fluctuation should be targeted to improve health outcomes. Intriguingly, higher variability in blood pressure [9–13], blood glucose [14], or body weight [15] was also associated with mild cognitive impairment, Alzheimer’s disease, and dementia, suggesting a new avenue of risk modification.

Metabolic risk factors are likely to cluster and influence interactively, resulting in a greater impact on an individual’s health status [16]. However, the composite effect of the variability of metabolic parameters on the risk of dementia has not been studied previously and remains to be better understood. In this study, we examined the prognostic significance of increased variability of BP, glucose, total cholesterol (TC), and body mass index (BMI) on dementia using a large nationwide population-based cohort involving nearly 3 million Koreans.

## Methods

### Data source and study population

The National Health Insurance System (NHIS) is a single-payer organization, managed by the government, to which all residents in Korea subscribe. Because it has adopted a fee-for-service system to pay healthcare providers, the NHIS obtains varied information which represents the entire Korean population. The database (DB) contains a qualification DB (e.g., age, sex, income, region, and type of eligibility), a claim DB (general information on specification, consultation statements, diagnosis statements defined by the International Classification of Disease 10th revision (ICD-10), and prescription statements), a health check-up DB, and death information. Enrollees in the NHIS are recommended to undergo a standardized medical examination at least every 2 years. Details on the DB are described elsewhere [17, 18].

**Fig. 1:**
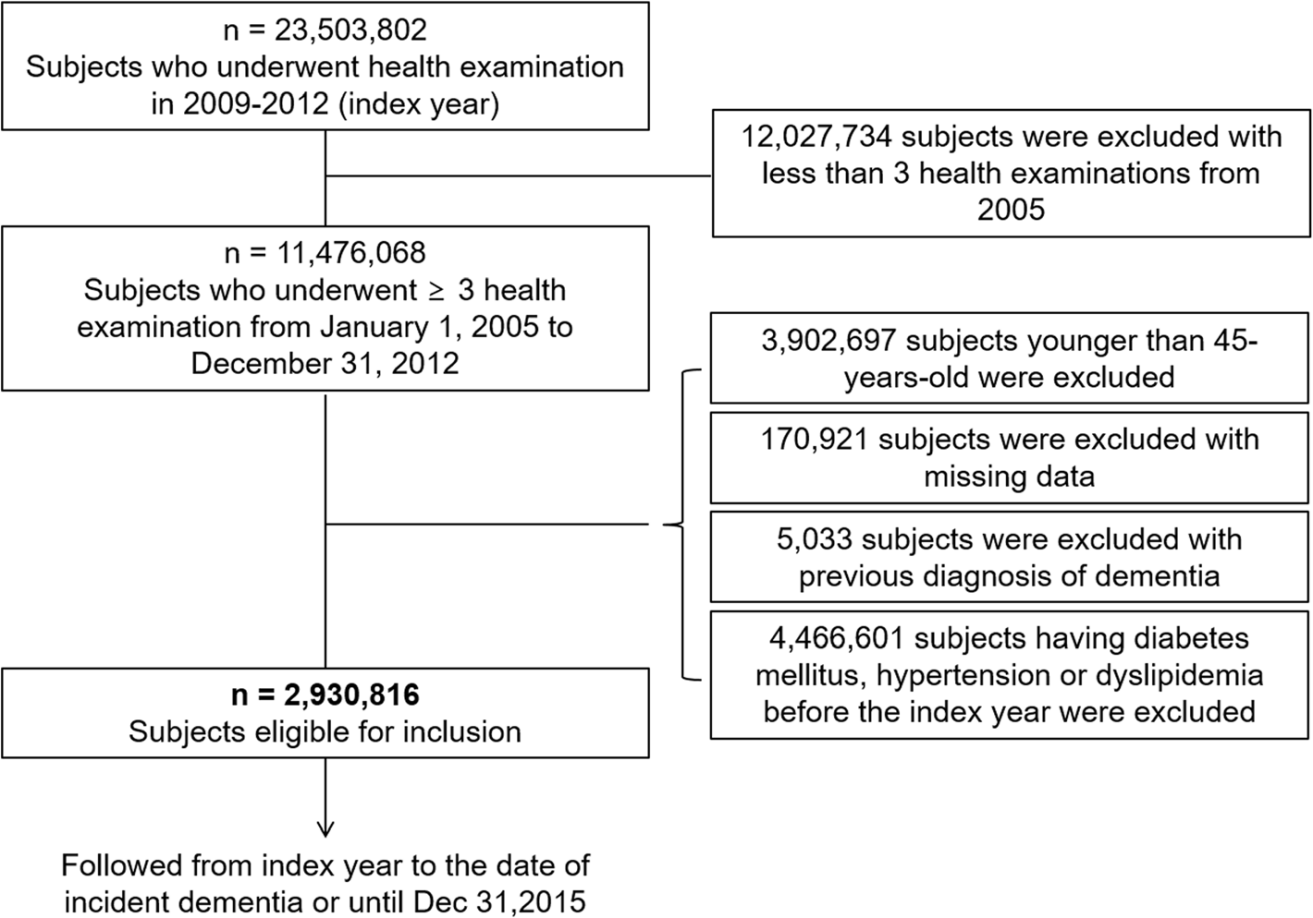
Flow chart of the study population

Among 23,503,802 subjects who underwent health examinations between 2009 and 2012 (index year), we excluded subjects who had fewer than three health examinations from 2005 (*n* = 12,027,734), were younger than 45 years old (*n* = 3,902,697), had data missing for at least one variable (*n* = 170,921), and had a previous diagnosis of dementia (*n* = 5033). Because the presence of metabolic diseases or administration of medications might artificially influence the variability of metabolic parameters, those who had a history of hypertension (at least one claim per year under ICD-10 codes I10–I13 or I15 and at least one claim per year for the prescription of antihypertensive agents, or systolic/diastolic BP ≥ 140/90 mmHg), diabetes mellitus (at least one claim per year under ICD-10 codes E10–E14 and at least one claim per year for the prescription of antidiabetic medication, or fasting glucose level ≥ 126 mg/dl), or dyslipidemia (at least one claim per year under ICD-10 code E78 and at least one claim per year for the prescription of a lipid-lowering agent, or TC ≥ 240 mg/dl) before the index year (*n* = 4,466,601) were also excluded from the main analyses. Ultimately, the study population consisted of 2,930,816 subjects (Fig. 1). The number of health examinations per subject was three (*n* = 2,192,400; 74.8%), four (*n* = 305,650; 10.4%), or five (*n* = 432,766; 14.8%). In order to examine whether our findings are reproducible in subjects having diabetes mellitus, hypertension, or dyslipidemia, additional analysis was performed in the subjects already having metabolic diseases and in the total population. This study was approved by the Institutional Review Board of Seoul St. Mary’s Hospital (No. KC17ZESI0505). Informed consent was not obtained because anonymous and de-identified information was used for the analysis.

### Measurements and definitions

BMI was calculated as a subject’s weight in kilograms divided by the square of the subject’s height in meters, and obesity was defined as BMI ≥ 25 kg/m^2^. Information on smoking history and alcohol consumption was obtained by questionnaire. Regular exercise was defined as performing more than 30 min of moderate physical activity at least five times per week or more than 20 min of strenuous physical activity at least three times per week. Income level was dichotomized at the lower 25%. Blood samples for the measurement of serum glucose and lipid levels were drawn after an overnight fast. Hospitals wherein these health examinations were performed were certified by the NHIS and subjected to regular quality control.

### Definition of variability indices

Variability in each parameter was defined as variability in their values measured by health examinations. Three indices of variability were used: coefficient of variation (CV), standard deviation (SD), and variability independent of the mean (VIM). The VIM was calculated as 100 × SD / mean^*β*^, where *β* is the regression coefficient, based on the natural logarithm of the SD over the natural logarithm of the mean. High variability was defined as values in the highest quartile for each parameter.

### Study outcomes and follow-up

The end point of the study was newly diagnosed dementia, which was further classified as Alzheimer’s disease, vascular dementia, or other dementia. The definition of these diseases was based on the recording of relevant ICD-10 codes (F00 or G30 for Alzheimer’s disease; F01 for vascular dementia; and F02, F03, or G31 for other dementia) and the prescription of medication for dementia (rivastigmine, galantamine, memantine, or donepezil). When both codes for Alzheimer’s disease and vascular dementia were recorded, we followed the principal diagnosis. If both were in the additional diagnosis up to the second claim DB, the subject was classified as other dementia. The study population was followed from baseline to the date of incident dementia or until December 31, 2015, whichever came first.

### Statistical analysis

Baseline characteristics are presented as the mean ± SD or number (percentage). Participants were classified into five groups according to the number of parameters with high variability. The incidence rate of dementia was calculated by dividing the number of incident cases by the total follow-up duration (person-years). The cumulative incidence of outcomes according to the number of parameters with high variability was calculated using Kaplan–Meier curves, and the log-rank test was performed to analyze differences among the groups. Hazard ratio (HR) and 95% confidence interval (CI) values were analyzed using the Cox proportional hazards model. The proportional hazards assumption was evaluated by the Schoenfeld Residuals Test with the logarithm of the cumulative hazards function based on Kaplan–Meier estimates for quartile groups of variability or groups based on the number of parameters with high variability. There was no significant departure from proportionality in hazards over time. The multivariable-adjusted proportional hazards model was applied: model 1 was adjusted for age, sex, smoking, alcohol consumption, regular exercise, and income; and model 2 was further adjusted for baseline glucose, systolic BP, TC, and BMI. Because an event of mortality could compete with our outcome of interest, we also performed competing risk analysis using a subdistribution hazards model [19, 20]. To account for the possible effects of metabolic status before the index year, we also tested the Cox proportional hazards model using mean values of glucose, systolic BP, TC, and BMI instead of baseline values. Sensitivity analyses were also performed, excluding subjects with end points occurring within 3 years of follow-up, or excluding subjects with ischemic heart disease (ICD-10 codes I20–I25), stroke (ICD-10 codes I63–I64), depression (ICD-10 codes F32–F33), head injury (ICD-10 codes S00–S09), Parkinson’s disease (ICD-10 codes G00–G22), and HIV infection (ICD-10 codes B20–B24). In addition, analyses using another criterion for high variability (being > 1 SD) and using a weighted variability score (variability score corrected for the strength of the association of each metabolic parameter with the risk of dementia) were performed. In consideration of the possible influence of incident diabetes, hypertension, or dyslipidemia during follow-up on the variability of metabolic parameters, analysis censoring these subjects was performed. The potential effect modification by age, sex, and obesity was evaluated through the stratified analysis and interaction testing using a likelihood ratio test. Statistical analyses were performed using SAS version 9.4 (SAS Institute Inc., Cary, NC, USA), and *P* < 0.05 was considered to indicate statistical significance.

## Results

### Study subject characteristics

The proportions of participants having zero, one, two, three, or four parameters of high variability (measured using the CV) were 33.7, 39.5, 20.6, 5.6, and 0.7%, respectively. The characteristics of subjects by the number of parameters with high variability are presented in Table 1. Subjects with more parameters of high variability were older, were less obese, and had higher BP and fasting glucose. The TC levels were approximately 197 mg/dl in all groups. The proportions of current smokers, heavy drinkers, and subjects with low income were higher in the groups with more parameters of high variability. Similar patterns of subject characteristics were noted when the variability was calculated as the SD and VIM (data not shown).

**Table 1:** Baseline characteristics of subjects according to number of parameters with high variability measured as coefficient of variation

Characteristic	0 parameters (*n* = 988,490)	1 parameter (*n* = 1,156,635)	2 parameters (*n* = 602,366)	3 parameters (*n* = 163,699)	4 parameters (*n* = 19,626)
Age (years)	53.9 ± 7.3	54.5 ± 7.8	55.4 ± 8.5	56.5 ± 9.2	57.7 ± 9.8
Sex (male)	526,920 (53.3)	578,516 (50.0)	291,666 (48.4)	79,928 (48.8)	9863 (50.3)
Height (cm)	162.9 ± 8.4	162.0 ± 8.4	161.3 ± 8.5	160.7 ± 8.5	160.4 ± 8.7
Weight (kg)	62.3 ± 9.8	61.3 ± 9.8	60.4 ± 9.9	59.5 ± 9.9	58.6 ± 10.0
Body mass index (kg/m^2^)					
Baseline	23.5 ± 2.6	23.5 ± 2.7	23.4 ± 2.8	23.3 ± 3.0	23.1 ± 3.1
Mean	23.4 ± 2.6	23.3 ± 2.6	23.2 ± 2.7	23.0 ± 2.7	22.8 ± 2.8
CV	2.02 ± 0.87	2.87 ± 1.95	3.82 ± 2.43	4.83 ± 2.68	5.82 ± 2.66
SD	0.47 ± 0.21	0.67 ± 0.46	0.88 ± 0.58	1.11 ± 0.64	1.33 ± 0.65
VIM	0.47 ± 0.20	0.66 ± 0.45	0.88 ± 0.56	1.11 ± 0.62	1.34 ± 0.61
Systolic BP (mmHg)					
Baseline	120.6 ± 11.3	120.9 ± 12.5	121.4 ± 13.5	121.9 ± 14.6	122.5 ± 15.8
Mean	119.1 ± 9.8	119.0 ± 10.0	119.1 ± 10.2	119.4 ± 10.5	120.0 ± 11.0
CV	5.45 ± 2.31	7.26 ± 3.81	8.92 ± 4.27	10.69 ± 4.16	12.81 ± 3.05
SD	6.48 ± 2.79	8.61 ± 4.58	10.61 ± 5.20	12.78 ± 5.19	15.42 ± 4.16
VIM	6.43 ± 2.73	8.57 ± 4.50	10.54 ± 5.06	12.65 ± 4.94	15.18 ± 3.69
Diastolic BP (mmHg)	75.8 ± 8.4	75.8 ± 8.7	75.9 ± 9.0	76.0 ± 9.4	76.2 ± 9.9
Fasting glucose (mg/dl)					
Baseline	94.3 ± 10.1	95.4 ± 12.8	96.6 ± 14.9	98.0 ± 16.7	99.5 ± 18.5
Mean	92.3 ± 8.5	92.5 ± 9.2	93.0 ± 9.9	93.7 ± 10.6	94.8 ± 11.6
CV	6.54 ± 2.83	9.20 ± 5.74	11.79 ± 6.81	14.47 ± 7.00	17.38 ± 6.28
SD	6.02 ± 2.67	8.61 ± 6.06	11.16 ± 7.57	13.83 ± 8.31	16.81 ± 8.48
VIM	5.89 ± 2.80	8.11 ± 4.79	10.23 ± 5.44	12.37 ± 5.34	14.62 ± 4.33
Total cholesterol (mg/dl)					
Baseline	197.0 ± 27.4	197.3 ± 29.1	197.5 ± 31.1	197.4 ± 33.4	196.5 ± 35.5
Mean	192.5 ± 24.8	191.2 ± 24.9	189.9 ± 25.1	188.7 ± 25.5	187.1 ± 25.9
CV	6.70 ± 2.75	8.94 ± 4.81	11.48 ± 5.66	14.12 ± 5.61	16.64 ± 4.65
SD	12.88 ± 5.54	17.00 ± 9.31	21.69 ± 10.98	26.54 ± 11.05	31.05 ± 9.51
VIM	12.49 ± 5.13	16.62 ± 8.93	21.33 ± 10.50	26.22 ± 10.39	30.86 ± 8.59
Log triglycerides	4.7 ± 0.5	4.6 ± 0.5	4.7 ± 0.5	4.7 ± 0.5	4.7 ± 0.5
HDL-cholesterol (mg/dl)	55.1 ± 17.9	55.4 ± 18.7	55.5 ± 19.2	55.6 ± 20.6	55.6 ± 21.8
LDL-cholesterol (mg/dl)	115.4 ± 35.3	114.0 ± 36.3	112.4 ± 37.5	110.3 ± 37.4	108.0 ± 41.8
Smoking					
None	627,703 (63.5)	750,500 (64.9)	393,231 (65.3)	105,325 (64.3)	12,331 (62.8)
Ex-smoker	177,795 (18.0)	184,318 (15.9)	89,071 (14.8)	23,457 (14.3)	2844 (14.5)
Current smoker	182,992 (18.5)	221,817 (19.2)	120,064 (19.9)	34,917 (21.3)	4451 (22.7)
Alcohol consumption					
None	562,435 (56.9)	687,756 (59.5)	367,873 (61.1)	101,334 (61.9)	12,340 (62.9)
< 30 g/day	369,632 (37.4)	402,512 (34.8)	198,101 (32.9)	51,318 (31.3)	5820 (29.7)
≥ 30 g/day	56,423 (5.7)	66,367 (5.7)	36,392 (6.0)	11,047 (6.7)	1466 (7.5)
Regular exercise	223,139 (22.6)	249,901 (21.6)	124,580 (20.7)	32,385 (19.8)	3674 (18.7)
Income (lower 25%)	181,845 (18.4)	240,264 (20.8)	135,512 (22.5)	39,191 (23.9)	4816 (24.5)
Ischemic heart disease	12,698 (1.3)	16,630 (1.4)	9659 (1.6)	3083 (1.9)	393 (2.0)
Stroke	4093 (0.4)	5982 (0.5)	3764 (0.6)	1206 (0.7)	198 (1.0)
Depression	25,577 (2.6)	36,391 (3.1)	22,900 (3.8)	7352 (4.5)	1014 (5.2)

Data expressed as mean ± standard deviation or *n* (%)

*P* for trend values <  0.001 for all variables

*BP* blood pressure, *CV* coefficient of variation, *HDL* high-density lipoprotein, *LDL* low-density lipoprotein, *SD* standard deviation, *VIM* variability independent of the mean

During a median (5–95%) follow-up of 5.5 (3.2–6.8) years, 32,901 (1.12%) participants developed dementia. Among them, 24,486 (74.4%) and 3629 (11.0%) cases were attributable to Alzheimer’s disease and vascular dementia, respectively. Subjects with incident dementia were older, were more likely to be female, were less obese, and had higher systolic BP and lower total cholesterol levels compared to the subjects without dementia. They also had higher variability indices for each of the metabolic parameters and were more likely to have undergone fewer health examinations (Additional file 1: Table S1).

### Variability of individual parameters and the risk of dementia

We first examined the effect of variability of individual metabolic parameters on the risk of incident dementia. The incidence rates for all-cause dementia in the highest CV quartile (Q4) groups of BMI, systolic BP, glucose, and TC variability were 133, 67, 31, and 36% higher compared to the lowest CV quartile (Q1) groups, respectively. An incrementally higher risk of all-cause dementia was observed with higher CV quartiles of BMI, systolic BP, glucose, and TC compared to the lowest quartile groups in model 1 (Additional file 1: Table S2). Similar findings were noted for the risk of Alzheimer’s disease, whereas the association between glucose variability and incident vascular dementia showed borderline significance. Even after further adjusting for baseline BMI, systolic BP, glucose, and TC levels (model 2), the association between variability and all-cause dementia remained significant (HR (95% CI): Q4 of BMI, 1.41 (1.36–1.45); Q4 of systolic BP, 1.15 (1.12–1.18); Q4 of glucose, 1.12 (1.08–1.15); Q4 of TC, 1.21 (1.18–1.25)) (Table 2). The association of dementia with diastolic BP variability was similar to that with systolic BP variability (data not shown). Additionally, little difference was observed for Alzheimer’s disease and vascular dementia between model 1 and model 2. The results were largely consistent when the variability was determined using the SD and the VIM (Additional file 1: Tables S3 and S4).

**Table 2:** Hazard ratios and 95% confidence intervals of all-cause dementia, Alzheimer’s disease, and vascular dementia by quartiles of metabolic parameter variability measured as coefficient of variation

	All-cause dementia			Alzheimer’s disease			Vascular dementia		
	Events (*n*)	Incidence rate (per 1000 person-years)	HR (95% CI)	Events (*n*)	Incidence rate (per 1000 person-years)	HR (95% CI)	Events (*n*)	Incidence rate (per 1000 person-years)	HR (95% CI)
Body mass index									
Q1	5818	1.50	1 (ref.)	4338	1.12	1 (ref.)	661	0.17	1 (ref.)
Q2	6264	1.59	**1.07 (1.03–1.11)**	4694	1.19	**1.07 (1.03–1.12)**	691	0.18	1.04 (0.94–1.16)
Q3	7443	1.90	**1.13 (1.09–1.17)**	5497	1.41	**1.11 (1.06–1.15)**	845	0.22	**1.17 (1.06–1.30)**
Q4	13,376	3.49	**1.41 (1.36–1.45)**	9957	2.60	**1.36 (1.31–1.41)**	1432	0.37	**1.51 (1.38–1.66)**
*P* for trend			< 0.001			< 0.001			< 0.001
Systolic blood pressure									
Q1	7058	1.81	1 (ref.)	5208	1.34	1 (ref.)	795	0.20	1 (ref.)
Q2	6586	1.71	1.02 (0.99–1.05)	4900	1.27	1.03 (0.99–1.07)	719	0.19	0.99 (0.89–1.09)
Q3	7607	1.94	**1.03 (1.00–1.07)**	5647	1.44	1.04 (1.00–1.08)	890	0.23	1.08 (0.98–1.19)
Q4	11,650	3.02	**1.15 (1.12–1.18)**	8731	2.26	**1.15 (1.11–1.19)**	1225	0.32	**1.16 (1.06–1.27)**
*P* for trend			< 0.001			< 0.001			< 0.001
Glucose									
Q1	7388	1.94	1 (ref.)	5449	1.43	1 (ref.)	821	0.22	1 (ref.)
Q2	7483	1.93	**1.04 (1.01–1.08)**	5568	1.43	**1.06 (1.02–1.10)**	837	0.22	1.04 (0.94–1.14)
Q3	8030	2.05	**1.06 (1.03–1.09)**	5983	1.53	**1.07 (1.03–1.11)**	914	0.23	1.08 (0.98–1.18)
Q4	10,000	2.54	**1.12 (1.08–1.15)**	7486	1.90	**1.13 (1.09–1.17)**	1057	0.27	1.08 (0.99–1.19)
*P* for trend			< 0.001			< 0.001			0.076
Total cholesterol									
Q1	7468	1.94	1 (ref.)	5589	1.45	1 (ref.)	805	0.21	1 (ref.)
Q2	7420	1.89	**1.07 (1.03–1.10)**	5477	1.40	**1.05 (1.01–1.09)**	832	0.21	1.09 (0.99–1.20)
Q3	7835	2.00	**1.09 (1.06–1.13)**	5896	1.51	**1.10 (1.06–1.14)**	873	0.22	**1.12 (1.02–1.23)**
Q4	10,178	2.64	**1.21 (1.18–1.25)**	7524	1.95	**1.19 (1.15–1.24)**	1119	0.29	**1.26 (1.15–1.38)**
*P* for trend			< 0.001			< 0.001			< 0.001

Adjusted for age, sex, smoking, alcohol consumption, regular exercise, income, glucose, systolic blood pressure, total cholesterol, and body mass index (model 2). Model 1 (adjusted for age, sex, smoking, alcohol consumption, regular exercise, and income) presented in Additional file 1: Table S2

*CI* confidence interval, *HR* hazard ratio, *Q1–Q4* quartiles 1 (lowest)–4 (highest), *ref.* reference

Bold data are data with statistical significance

### Number of parameters with high variability and the risk of dementia

**Fig. 2:**
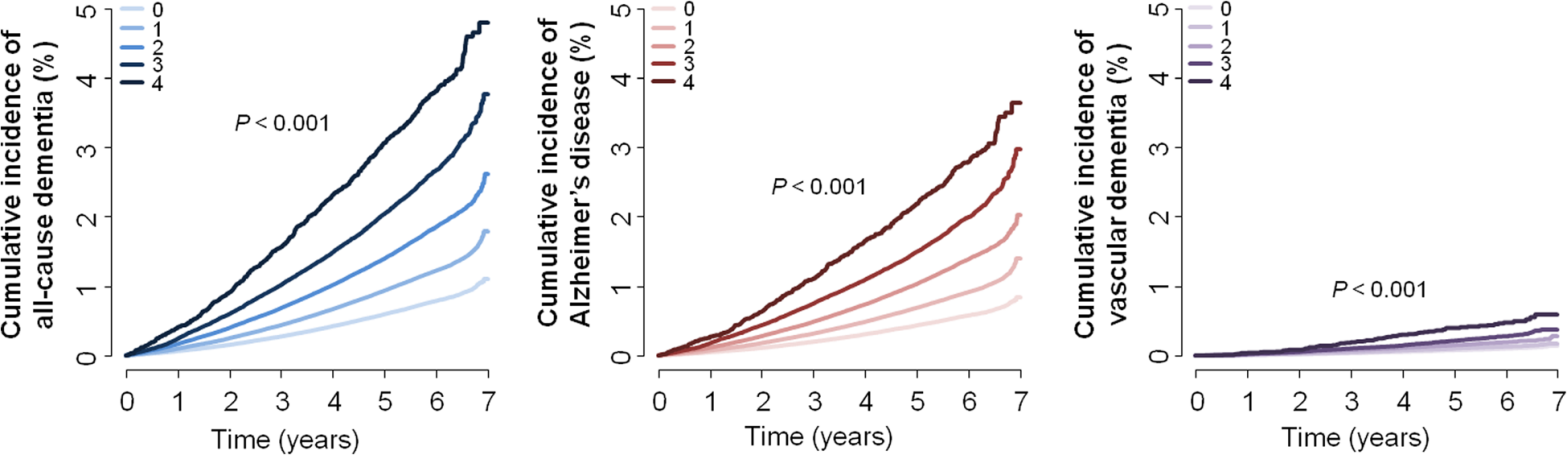
Cumulative incidence of all-cause dementia, Alzheimer’s disease, and vascular dementia according to number of metabolic parameters with high variability defined as highest quartile of coefficient of variation

Next, we explored the composite effect of metabolic parameter variability on the risk of incident dementia. An incrementally higher cumulative incidence and incidence rate of all-cause dementia, Alzheimer’s disease, and vascular dementia were noted with a higher number of parameters with high variability (Fig. 2, Table 3). After adjusting for possible confounding factors, the HR (95% CI) of all-cause dementia was 1.22 (1.19–1.26) for one parameter, 1.39 (1.35–1.43) for two parameters, 1.54 (1.48–1.60) for three parameters, and 1.73 (1.60–1.88) for four parameters compared with subjects having no parameters of high variability measured as the CV. This dose–response relationship was also observed for the risk of Alzheimer’s disease and vascular dementia. Competing risk analysis including mortality as a competing risk showed similar results. Selection of covariates by backward stepwise elimination did not change the results (Additional file 1: Table S5). The results were largely consistent when the variability was determined using the SD and the VIM (Additional file 1: Figures S1 and S2, Tables S6 and S7).

**Table 3:** Hazard ratios and 95% confidence intervals of all-cause dementia, Alzheimer’s disease, and vascular dementia by number of parameters with high variability measured as coefficient of variation

Number of parameters	Events (*n*)	Incidence rate (per 1000 person-years)	Model 1^a^	Model 2^b^	Competing risk analysis^c^
All-cause dementia					
0	6744	1.28	1 (ref.)	1 (ref.)	1 (ref.)
1	12,195	1.99	**1.23 (1.19–1.27)**	**1.22 (1.19–1.26)**	**1.22 (1.18–1.25)**
2	9528	3.00	**1.41 (1.37–1.46)**	**1.39 (1.35–1.43)**	**1.38 (1.33–1.42)**
3	3783	4.40	**1.58 (1.51–1.64)**	**1.54 (1.48–1.60)**	**1.50 (1.43–1.56)**
4	651	6.38	**1.79 (1.65–1.94)**	**1.73 (1.60–1.88)**	**1.66 (1.53–1.81)**
*P* for trend			< 0.001	< 0.001	< 0.001
Alzheimer’s disease					
0	4952	0.94	1 (ref.)	1 (ref.)	1 (ref.)
1	9136	1.49	**1.24 (1.20–1.28)**	**1.23 (1.19–1.27)**	**1.22 (1.18–1.27)**
2	7106	2.24	**1.40 (1.35–1.45)**	**1.37 (1.32–1.42)**	**1.36 (1.31–1.41)**
3	2818	3.28	**1.55 (1.48–1.62)**	**1.50 (1.43–1.57)**	**1.46 (1.39–1.53)**
4	474	4.64	**1.71 (1.55–1.88)**	**1.65 (1.50–1.81)**	**1.58 (1.43–1.74)**
*P* for trend			< 0.001	< 0.001	< 0.001
Vascular dementia					
0	815	0.16	1 (ref.)	1 (ref.)	1 (ref.)
1	1350	0.22	**1.18 (1.09–1.29)**	**1.18 (1.08–1.29)**	**1.18 (1.08–1.29)**
2	990	0.31	**1.34 (1.22–1.47)**	**1.34 (1.22–1.47)**	**1.33 (1.21–1.46)**
3	393	0.46	**1.56 (1.38–1.77)**	**1.56 (1.38–1.76)**	**1.52 (1.35–1.72)**
4	81	0.79	**2.18 (1.73–2.74)**	**2.17 (1.72–2.73)**	**2.09 (1.66–2.64)**
*P* for trend			< 0.001	< 0.001	< 0.001

*ref.* reference

^a^Adjusted for age, sex, smoking, alcohol consumption, regular exercise, and income

^b^Adjusted for model 1 plus glucose, systolic blood pressure, total cholesterol, and body mass index

^c^Analysis using subdistribution hazards model, mortality considered a competing risk

Bold data are data with statistical significance

**Fig. 3:**
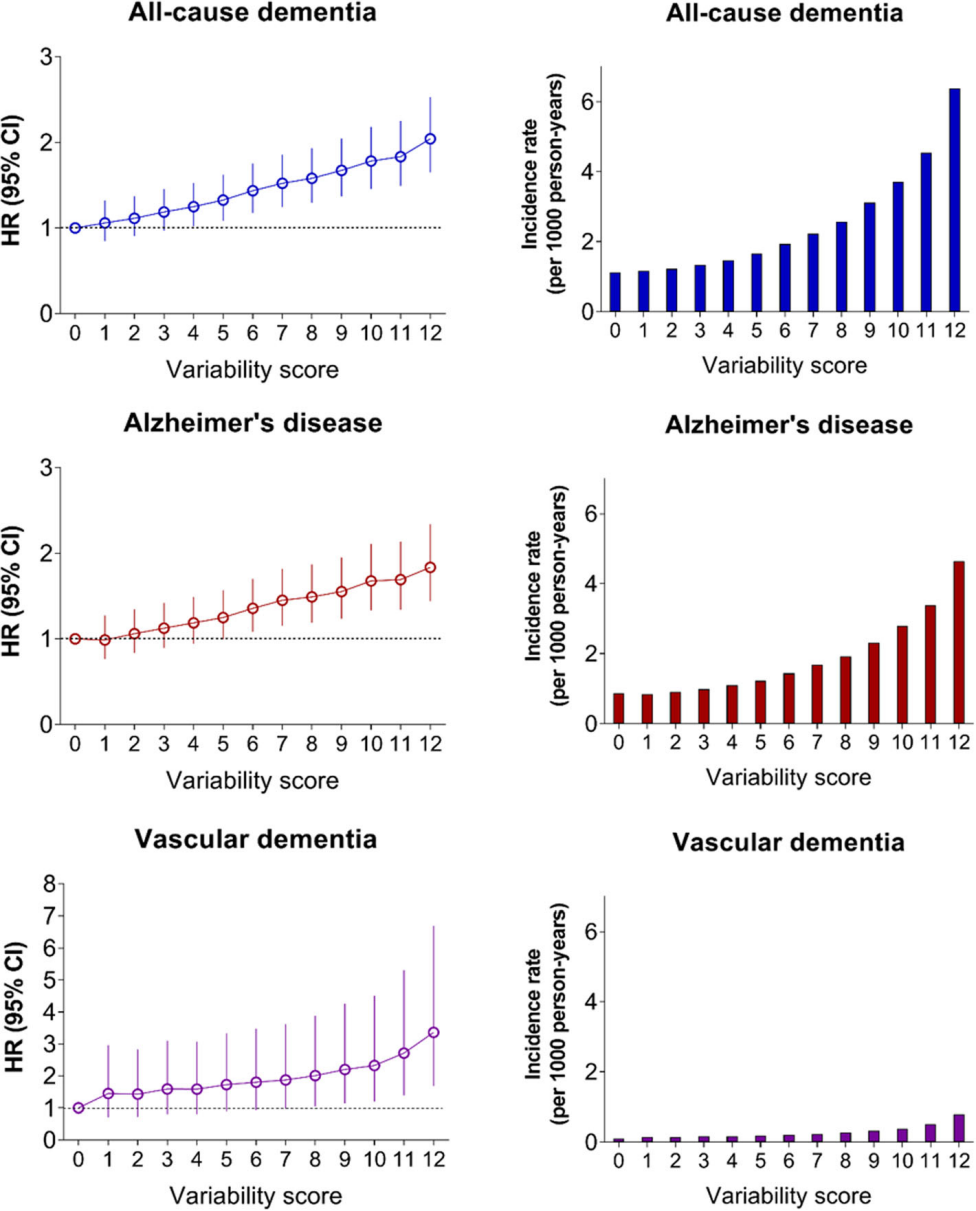
Incidence rate, hazard ratio (HR), and 95% confidence interval (CI) of all-cause dementia, Alzheimer’s disease, and vascular dementia according to variability score. 0 points assigned for Q1 (lowest quartile of variability), 1 point for Q2, 2 points for Q3, and 3 points for Q4 (highest quartile of variability) each for BP, glucose, cholesterol, and body mass index variability measured as coefficient of variation. Total score ranged from 0 to 12. Adjusted for age, sex, smoking, alcohol consumption, regular exercise, income, glucose, systolic blood pressure, total cholesterol, and body mass index

To evaluate the influence of lower quartile groups together, we defined a variability scoring system where 0 points were assigned for Q1, 1 point for Q2, 2 points for Q3, and 3 points for Q4 groups for each of four parameters. Therefore, the total score ranged from 0 to 12. It was clear that there is a positive linear association between the variability score and the incidence rate or risk of all-cause dementia, Alzheimer’s disease, and vascular dementia (*P* for trend < 0.001) (Fig. 3, Additional file 1: Figures S3 and S4).

### Sensitivity analysis

To account for the possibility of reverse causation, sensitivity analysis was performed excluding subjects with the occurrence of end points within 3 years of follow-up. Similar to the original analysis, an incrementally higher incidence rate and HR (95% CI) of all-cause dementia, Alzheimer’s disease, and vascular dementia were noted with a higher number of parameters with high variability (Additional file 1: Table S8). The results were nearly identical when mean levels of metabolic parameters were adjusted instead of baseline levels in the Cox proportional hazards model (data not shown). Excluding subjects with known risk factors of dementia including ischemic heart disease, stroke, depression, head injury, Parkinson’s disease, and HIV infection (Additional file 1: Table S9) or further adjusting for these diseases (Additional file 1: Table S10) did not attenuate the association between the number of parameters with high variability and outcomes. Applying a different criterion for high variability, being > 1 SD, resulted in similar observations (Additional file 1: Table S11). There was a graded association between the degree of variability and the risk of outcomes when the weighted variability score was used (Additional file 1: Table S12). During follow-up, 694,637 subjects (23.7% of the study population) developed diabetes, hypertension, or dyslipidemia. Analysis censoring these subjects showed similar results (Additional file 1: Table S13).

### Subgroup analysis

**Fig. 4:**
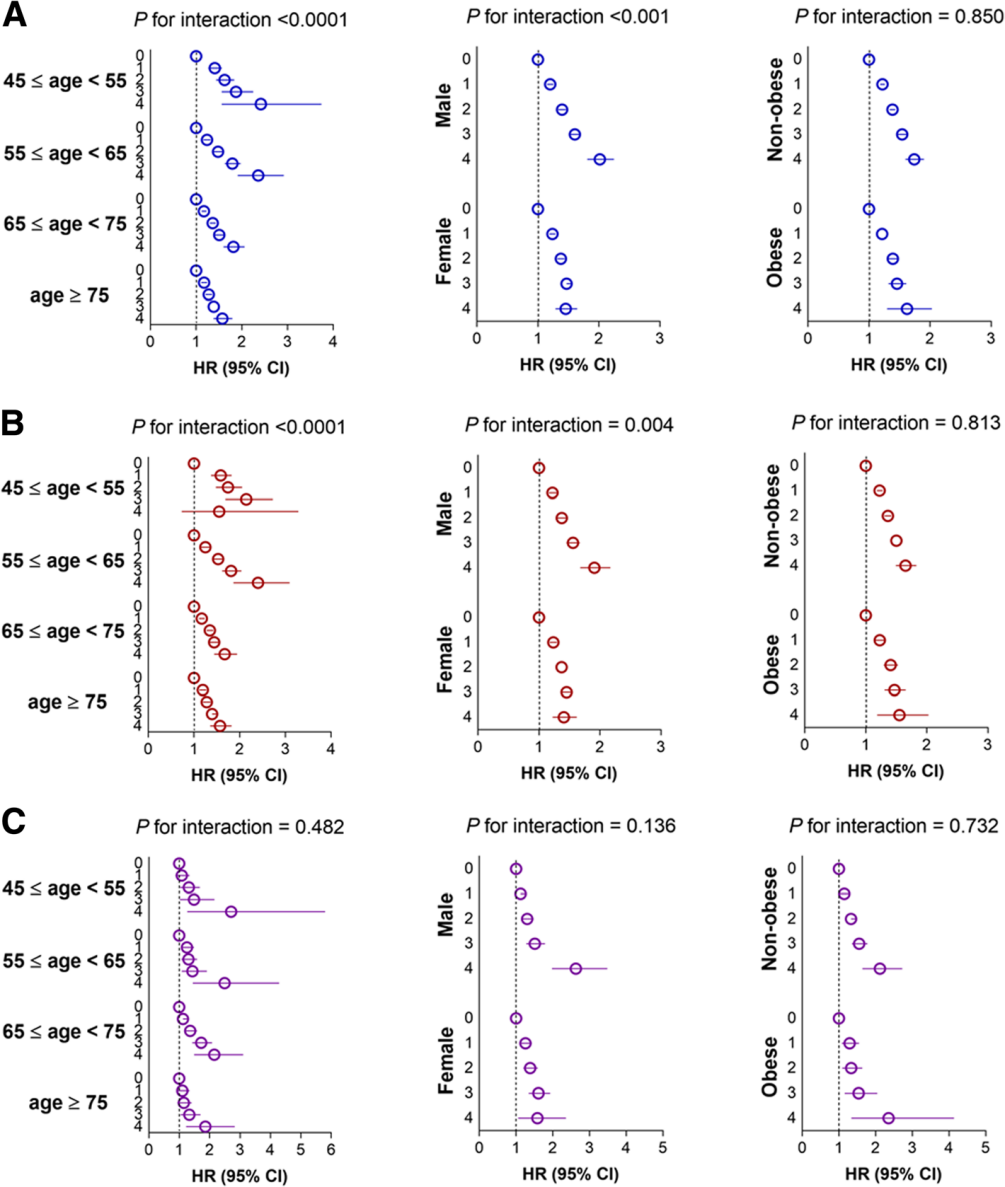
Hazard ratio (HR) and 95% confidence interval (CI) of all-cause dementia (**a**), Alzheimer’s disease (**b**), and vascular dementia (**c**) by number of metabolic parameters with high variability defined as highest quartile of coefficient of variation. Subgroup analyses according to age, sex, and presence or absence of obesity. Adjusted for age, sex, smoking, alcohol consumption, regular exercise, income, glucose, systolic blood pressure, total cholesterol, and body mass index

Stratified analysis by age, sex, and presence or absence of obesity was conducted. The significant association between the number of parameters with high variability and the risk of all-cause dementia, Alzheimer’s disease, and vascular dementia was present in all subgroups (Fig. 4). Higher adjusted HRs for all-cause dementia and Alzheimer’s disease were observed in younger aged (*P* for interaction < 0.001 for both outcomes) and male (*P* for interaction < 0.001 and 0.004, respectively) subgroups. However, there was no difference in the risk of vascular dementia according to subgroups. Additionally, nonobese and obese subjects showed similar risks of outcomes. These findings were in common with other indices of variability (Additional file 1: Figures S5 and S6).

### Analysis including subjects with known metabolic diseases

In line with the main analysis, significant association between the number of parameters with high variability and the risk of dementia was noted when a time-dependent Cox regression analysis was performed in the total population (including subjects with known diabetes, hypertension, or dyslipidemia) and using blood glucose, BP, and TC levels as time-dependent covariates. The HR (95% CI) was 1.74 (1.67–1.80) for all-cause dementia, 1.70 (1.63–1.78) for Alzheimer’s disease, and 1.77 (1.59–1.97) for vascular dementia when subjects having four parameters were compared with subjects having no parameters of high variability measured as the CV. Similar results were observed in the population having these metabolic diseases at baseline (Additional file 1: Table S14).

## Discussion

In this large-scale nationwide study of the general population, we examined the association between variability of metabolic parameters and the risk of developing dementia. Individuals with higher variability of each parameter (BMI, systolic BP, glucose, or total cholesterol) were at higher risk of future dementia. Of note, a composite effect of these parameters was evident, showing a linear association between the number of parameters with high variability and outcome measures. The results were consistent by various sensitivity analyses and in different subgroups, confirming that this relationship is widely applicable.

Hypertension, type 2 diabetes, and obesity are all well-established risk factors for dementia [2]. Because metabolic diseases also contribute to the progression from mild cognitive impairment to dementia [21], proper management of these conditions is considered an important therapeutic goal. Recently, in addition to having metabolic diseases, variability in BP, glucose, and BMI has gained much interest as a novel risk factor for cognitive decline and dementia. Among these, BP variability is the index most frequently studied and shows consistent results as an independent risk factor in an elderly population [9–13]. Visit-to-visit variability calculated from BP measured every 3 months or biennially was associated with impaired cognitive function or incident dementia independent of the average BP level [9, 11]. Similarly, variability from 24-h ambulatory BP monitoring or day-to-day home BP measurements for a median of 28 days was also an independent risk factor for the 5 years of the follow-up period [10, 12]. These data suggest that both short-term and long-term BP variability induces or reflects pathological conditions associated with cognitive decline and dementia. Because BP variability is also known to be related to mortality, coronary heart disease, stroke, and end-stage renal disease [3], the importance of stabilizing BP variability should be further emphasized. Glucose variability and HbA1c variability, reflecting short-term and long-term glycemic fluctuation, have been linked to various complications in both type 1 and type 2 diabetic patients [5, 22, 23]. Recently, a Taiwanese study reported that the CV of fasting plasma glucose and HbA1c were associated with an increased risk of Alzheimer’s disease independent of traditional risk factors in patients with type 2 diabetes [14]. Rawlings et al. [24] also showed that serum 1,5-anhydroglucitol levels, which reflect hyperglycemic peaks, measured in midlife are a risk factor for dementia and 20-year cognitive decline in participants with diabetes. However, whether this notion applies to nondiabetic subjects was unclear. The effect of body weight variability on dementia is controversial. While one study identified midlife body weight variability as a risk for late-life dementia [15], another study of elderly women showed no significant association after adjustment of covariates [25]. In addition, we and others suggested cholesterol variability as a risk factor for mortality, cardiovascular outcomes, and end-stage renal disease in the general population or patients with coronary heart disease [6, 26, 27], whereas it was unknown whether the risk of dementia is associated with high cholesterol variability. Importantly, these parameters are intercorrelated and likely to appear in clusters as a metabolic syndrome. Our study is the first to reveal a dose–response relationship between the number of metabolic parameters with high variability and the risk of all-cause dementia, Alzheimer’s disease, and vascular dementia.

While the exact mechanism for this phenomenon remains to be elucidated, several plausible explanations can be raised. First, hemodynamic instability leads to inflammation, endothelial dysfunction, and oxidative stress with consequent damage in brain structure and function [28, 29]. Therefore, fluctuations in BP can be detrimental to neuronal cells, leading to cell death and hippocampal atrophy by cerebral hypoperfusion and small vessel disease. Second, dementia is closely related to dysglycemia and insulin resistance in the central nervous system and is even referred to as type 3 diabetes [30, 31]. It has been shown that oscillating glucose has more deleterious effects than constant hyperglycemia on endothelial function, monocyte adhesion, and oxidative stress in humans and in-vitro studies [32, 33]. This could be associated with an overactivation of glycogen synthase kinase-3β and hyperphosphorylation of microtubule-associated protein tau resulting in disruption of neuronal function [34]. Third, higher cholesterol variability has been reported to be associated with lower cerebral flow and greater white matter hyperintensity load [35]. This could be mediated by endothelial dysfunction or plaque instability resulting from repeated fluctuations in the atherosclerotic plaque composition which may induce cerebrovascular damage. Fourth, body weight variability and associated dysfunctional production of certain hormones may negatively affect brain health [36]. For example, higher levels of leptin were associated with a lower risk of dementia [37].

An important question that can be raised is whether the variability in metabolic parameters is really a risk factor or is merely an indicator of increased risk for major health outcomes. Patients with cognitive decline might have difficulties in self-care, disease management, and low compliance to medications with consequent instability in several biological parameters, increasing the possibility of reverse causation. It is also suggested that central autonomic dysfunction may accompany dementia, which could contribute to worsening of variability in BP and glucose [38]. However, our data showed a strong correlation between variability indices and dementia with a dose–response relationship, were consistent with previous data, and were coherent with laboratory findings supporting the causal relationship [39, 40]. We also performed sensitivity analyses to strengthen the temporal relationship of the association, and confirmed similar results. To further dissect the causality issue, a prospective intervention controlling for the degree of variability would be informative, although these kinds of studies are difficult to perform in the real world. A study comparing the effect of repaglinide and glibenclamide, two different classes of antidiabetic agents, in type 2 diabetic patients showed that significant decline in the CV of postprandial plasma glucose and associated preservation of cognitive functioning was only observed in the repaglinide group [41]. Another study compared different antihypertensive classes, demonstrating that a reduced dementia risk associated with these agents was independent of BP variability [13]. This indirect evidence, although insufficient and controversial, suggests that stabilization of metabolic parameters by managing lifestyle behavior or using selected classes of medications could be a potential therapeutic target for the prevention of dementia.

We also showed that the association between metabolic parameter variability and all-cause dementia or Alzheimer’s disease was stronger in the younger population. It is likely that variability in metabolic parameters may contribute less in elderly people than in younger people, because there are other multiple risk factors associated with aging which increase the risk of developing dementia.

This study has the strength of using a large-scale nationwide database representing the entire Korean population. Whereas other studies mostly focused on diseased patients, we excluded subjects with hypertension, diabetes mellitus, and dyslipidemia to exclude potential influences of medication compliance or the disease itself. Our data suggest that the variability of metabolic parameters is a significant risk factor for dementia, even in a relatively low-risk population. In addition, similar results were observed in subjects with these metabolic diseases. However, limitations should also be acknowledged. First, there might be discrepancies between the actual diagnosis and the information recorded in the claim database. Classification of specific dementia subtypes could sometimes be difficult in clinical practice, leading to inaccurate recording of the diagnosis. We tried to overcome this issue by combining the diagnosis statements and the prescription statements. Second, because of the lack of cognitive function testing or imaging data, the severity of dementia could not be assessed. It would be interesting to examine the relationship between the degree of variability and the rate of cognitive decline in the future. Third, it is possible that some of the unknown factors influencing the variability of metabolic parameters might moderate the dementia risk. We tried to adjust covariates or exclude subjects with known risk factors to minimize this possibility. However, the effect of genetic factors still remains to be elucidated. Fourth, because the measurement of high-density and low-density lipoprotein cholesterol in the health examination of the NHIS was started in 2008, the association between variability of these parameters and dementia could not be explored due to an insufficient follow-up period. Fifth, exclusion of subjects with fewer than three health examinations or missing data might be a source of selection bias because employee subscribers with lower risk of dementia were more likely to participate in the regular health check-up [17]. Lastly, because the optimal method of calculating variability is unknown, the results might differ according to the definition of variability. Whether short-term (day-to-day) vs long-term (visit-to-visit) or mid-life vs recent variability best reflects the risk of dementia remains to be elucidated.

## Conclusion

We suggest that variability in metabolic parameters is an independent predictor for developing dementia. These findings indicate that reducing variability of metabolic parameters would be important in promoting resilience and preserving cognitive reserve in the general population. Further studies seeking optimal treatment modalities for the control and stabilization of metabolic parameters are warranted.

## Additional file

Additional file 1: **Table S1.** Characteristics of subjects according to incident dementia. **Tables S2–S4.** HR (95% CI) of all-cause dementia, Alzheimer’s disease, and vascular dementia by quartiles of metabolic parameter variability measured as CV (model 1), as SD, and as VIM. **Tables S5–S11.** HR (95% CI) of all-cause dementia, Alzheimer’s disease, and vascular dementia by number of parameters with high variability with covariates selected by backward stepwise elimination, measured as SD, measured as VIM, by sensitivity analysis excluding subjects with the occurrence of end points within 3 years of follow-up, by sensitivity analysis excluding diseases known as a risk factor of dementia, by further adjusting for diseases known as a risk factor of dementia, and with high variability defined as > 1 SD. **Table S12.** HR (95% CI) of all-cause dementia, Alzheimer’s disease, and vascular dementia by weighted variability score. **Tables S13–S14.** HR (95% CI) of all-cause dementia, Alzheimer’s disease, and vascular dementia by number of parameters with high variability measured as CV by sensitivity analysis (censoring cases with incident diabetes mellitus, hypertension, or dyslipidemia during the follow-up period) and in total population or subjects with metabolic disease at baseline. **Figures S1–S2.** Cumulative incidence of all-cause dementia, Alzheimer’s disease, and vascular dementia according to number of metabolic parameters with high variability defined as highest quartile of SD and of VIM. **Figures S3–S4.** Incidence rate, HR (95% CI) of all-cause dementia, Alzheimer’s disease, and vascular dementia according to variability score as SD and as VIM. **Figures S5–S6.** HR (95% CI) of all-cause dementia (**A**), Alzheimer’s disease (**B**), and vascular dementia (**C)** by number of metabolic parameters with high variability defined as highest quartile of SD and VIM (subgroup analyses according to age, sex, and presence or absence of obesity) (DOC 1137 kb)

## Abbreviations

BMI: Body mass index
BP: Blood pressure
CI: Confidence interval
CV: Coefficient of variation
DB: Database
HR: Hazard ratio
NHIS: National Health Insurance System
SD: Standard deviation
TC: Total cholesterol
VIM: Variability independent of the mean
