# Supplementary material for: Variability in metabolic parameters and risk of dementia: a nationwide population-based study
Source: Alzheimers Res Ther. 2018 Oct 27;10:110. doi: 10.1186/s13195-018-0442-3 (PMC6204276; doi:10.1186/s13195-018-0442-3)
Supplement: Supplementary file 1 — Table S1. Characteristics of subjects according to incident dementia. Tables S2–S4. HR (95% CI) of all-cause dementia, Alzheimer’s disease, and vascular dementia by quartiles of metabolic parameter variability measured as CV (model 1), as SD, and as VIM. Tables S5–S11. HR (95% CI) of all-cause dementia, Alzheimer’s disease, and vascular dementia by number of parameters with high variability with covariates selected by backward stepwise elimination, measured as SD, measured as VIM, by sensitivity analysis excluding subjects with the occurrence of end points within 3 years of follow-up, by sensitivity analysis excluding diseases known as a risk factor of dementia, by further adjusting for diseases known as a risk factor of dementia, and with high variability defined as > 1 SD. Table S12. HR (95% CI) of all-cause dementia, Alzheimer’s disease, and vascular dementia by weighted variability score. Tables S13–S14. HR (95% CI) of all-cause dementia, Alzheimer’s disease, and vascular dementia by number of parameters with high variability measured as CV by sensitivity analysis (censoring cases with incident diabetes mellitus, hypertension, or dyslipidemia during the follow-up period) and in total population or subjects with metabolic disease at baseline. Figures S1–S2. Cumulative incidence of all-cause dementia, Alzheimer’s disease, and vascular dementia according to number of metabolic parameters with high variability defined as highest quartile of SD and of VIM. Figures S3–S4. Incidence rate, HR (95% CI) of all-cause dementia, Alzheimer’s disease, and vascular dementia according to variability score as SD and as VIM. Figures S5–S6. HR (95% CI) of all-cause dementia (A), Alzheimer’s disease (B), and vascular dementia (C) by number of metabolic parameters with high variability defined as highest quartile of SD and VIM (subgroup analyses according to age, sex, and presence or absence of obesity) (DOC 1137 kb) [file 13195_2018_442_MOESM1_ESM.doc]

**Table S1** Characteristics of subjects according to incident dementia

|  | No dementia  (n = 2897915) | Dementia  (n = 32901) | *P* |
| --- | --- | --- | --- |
| Age (years) | 54.4 ± 7.8 | 70.2 ± 8.8 | < 0.001 |
| Sex (male) | 1471174 (50.8) | 15719 (47.8) | < 0.001 |
| Height (cm) | 162.1 ± 8.4 | 156.8 ± 9.0 | < 0.001 |
| Weight (kg) | 61.4 ± 9.8 | 55.3 ± 9.6 | < 0.001 |
| Body mass index (kg/m^2^) |  |  |  |
| Baseline | 23.5 ± 2.7 | 22.6 ± 3.0 | < 0.001 |
| Mean | 23.3 ± 2.6 | 22.5 ± 2.8 | < 0.001 |
| CV | 2.90 ± 2.01 | 3.81 ± 2.95 | < 0.001 |
| SD | 0.67 ± 0.48 | 0.85 ± 0.67 | < 0.001 |
| VIM | 0.67 ± 0.46 | 0.88 ± 0.68 | < 0.001 |
| Systolic BP (mmHg) |  |  |  |
| Baseline | 121.0 ± 12.5 | 122.8 ± 12.8 | < 0.001 |
| Mean | 119.0 ± 10.0 | 122.1 ± 10.3 | < 0.001 |
| CV | 7.20 ± 3.84 | 8.37 ± 4.71 | < 0.001 |
| SD | 8.56 ± 4.64 | 10.25 ± 5.96 | < 0.001 |
| VIM | 8.51 ± 4.54 | 9.97 ± 5.64 | < 0.001 |
| Diastolic BP (mmHg) | 75.8 ± 8.7 | 75.5 ± 8.5 | < 0.001 |
| Fasting glucose (mg/dL) |  |  |  |
| Baseline | 95.5 ± 12.8 | 95.5 ± 12.7 | 0.405 |
| Mean | 92.6 ± 9.2 | 93.3 ± 9.7 | < 0.001 |
| CV | 9.17 ± 5.82 | 10.02 ± 6.53 | < 0.001 |
| SD | 8.60 ± 6.20 | 9.51 ± 7.16 | < 0.001 |
| VIM | 8.07 ± 4.83 | 8.64 ± 5.22 | < 0.001 |
| Total cholesterol (mg/dL) |  |  |  |
| Baseline | 197.3 ± 29.3 | 193.2 ± 29.8 | < 0.001 |
| Mean | 191.2 ± 25.0 | 189.8 ± 25.7 | < 0.001 |
| CV | 9.04 ± 5.00 | 9.80 ± 5.59 | < 0.001 |
| SD | 17.19 ± 9.65 | 18.47 ± 10.65 | < 0.001 |
| VIM | 16.81 ± 9.28 | 18.21 ± 10.35 | < 0.001 |
| Log Triglycerides | 4.7 ± 0.5 | 4.7 ± 0.5 | 0.328 |
| HDL-cholesterol (mg/dL) | 55.3 ± 18.6 | 54.7 ± 23.8 | < 0.001 |
| LDL-cholesterol (mg/dL) | 113.9 ± 36.4 | 111.8 ± 35.1 | < 0.001 |
| Smoking |  |  | < 0.001 |
| None | 1865819 (64.4) | 23271 (70.7) |  |
| Ex-smoker | 473107 (16.3) | 4378 (13.3) |  |
| Current smoker | 558989 (19.3) | 5252 (16.0) |  |
| Alcohol consumption |  |  | < 0.001 |
| None | 1707206 (58.9) | 24532 (74.6) |  |
| < 30 g/day | 1020727 (35.2) | 6656 (20.2) |  |
| ≥ 30 g/day | 169982 (5.9) | 1713 (5.2) |  |
| Regular exercise | 628162 (21.7) | 5517 (16.8) | < 0.001 |
| Income (lower 25%) | 594899 (20.5) | 6729 (20.5) | 0.734 |
| Ischemic heart disease | 41204 (1.4) | 1259 (3.8) | < 0.001 |
| Stroke | 14090 (0.5) | 1153 (3.5) | < 0.001 |
| Depression | 90045 (3.1) | 3189 (9.7) | < 0.001 |
| Number of health examinations |  |  | < 0.001 |
| 3 | 2161958 (74.6) | 30442 (92.5) |  |
| 4 | 304288 (10.5) | 1362 (4.1) |  |
| 5 | 431669 (14.9) | 1097 (3.3) |  |

Data are expressed as the means ± SD, or n (%).

Abbreviations: BP, blood pressure; CV, coefficient of variation; HDL, high-density lipoprotein; LDL, low-density lipoprotein; SD, standard deviation; VIM, variability independent of the mean.

**Table S2 Hazard ratios and 95% confidence intervals of all-cause dementia, Alzheimer’s disease and vascular dementia by quartiles of metabolic parameter variability measured as coefficient of variation (model 1)**

|  | **All-cause dementia** | | | **Alzheimer’s disease** | | | **Vascular dementia** | | |
| --- | --- | --- | --- | --- | --- | --- | --- | --- | --- |
|  | Events (n) | Incidence rate (per 1000  person-years) | HR (95% CI) | Events (n) | Incidence rate (per 1000  person-years) | HR (95% CI) | Events (n) | Incidence rate (per 1000  person-years) | HR (95% CI) |
| **Body mass index** | | | | | | | | | |
| Q1 | 5818 | 1.50 | 1 (ref) | 4338 | 1.12 | 1 (ref) | 661 | 0.17 | 1 (ref) |
| Q2 | 6264 | 1.59 | **1.07 (1.03-1.11)** | 4694 | 1.19 | **1.07 (1.03-1.12)** | 691 | 0.18 | 1.04 (0.94-1.16) |
| Q3 | 7443 | 1.90 | **1.14 (1.10-1.18)** | 5497 | 1.41 | **1.12 (1.07-1.16)** | 845 | 0.22 | **1.17 (1.06-1.30)** |
| Q4 | 13376 | 3.49 | **1.43 (1.39-1.47)** | 9957 | 2.60 | **1.39 (1.34-1.44)** | 1432 | 0.37 | **1.51 (1.38-1.66)** |
| *P* for trend | |  | < 0.001 |  |  | < 0.001 |  |  | < 0.001 |
| **Systolic blood pressure** | | | | | | | | | |
| Q1 | 7058 | 1.81 | 1 (ref) | 5208 | 1.34 | 1 (ref) | 795 | 0.20 | 1 (ref) |
| Q2 | 6586 | 1.71 | 1.02 (0.99-1.06) | 4900 | 1.27 | 1.04 (1.00-1.08) | 719 | 0.19 | 0.98 (0.88-1.08) |
| Q3 | 7607 | 1.94 | **1.04 (1.00-1.07)** | 5647 | 1.44 | **1.04 (1.00-1.08)** | 890 | 0.23 | 1.08 (0.98-1.19) |
| Q4 | 11650 | 3.02 | **1.16 (1.13-1.19)** | 8731 | 2.26 | **1.16 (1.12-1.20)** | 1225 | 0.32 | **1.16 (1.06-1.26)** |
| *P* for trend | |  | < 0.001 |  |  | < 0.001 |  |  | < 0.001 |
| **Glucose** | | | | | | | | | |
| Q1 | 7388 | 1.94 | 1 (ref) | 5449 | 1.43 | 1 (ref) | 821 | 0.22 | 1 (ref) |
| Q2 | 7483 | 1.93 | **1.05 (1.01-1.08)** | 5568 | 1.43 | **1.06 (1.02-1.10)** | 837 | 0.22 | 1.04 (0.94-1.14) |
| Q3 | 8030 | 2.05 | **1.07 (1.03-1.10)** | 5983 | 1.53 | **1.08 (1.04-1.12)** | 914 | 0.23 | 1.08 (0.98-1.19) |
| Q4 | 10000 | 2.54 | **1.14 (1.11-1.18)** | 7486 | 1.90 | **1.15 (1.11-1.19)** | 1057 | 0.27 | 1.09 (0.99-1.19) |
| *P* for trend | |  | < 0.001 |  |  | < 0.001 |  |  | 0.056 |
| **Total cholesterol** | | | | | | | | | |
| Q1 | 7468 | 1.94 | 1 (ref) | 5589 | 1.45 | 1 (ref) | 805 | 0.21 | 1 (ref) |
| Q2 | 7420 | 1.89 | **1.07 (1.03-1.10)** | 5477 | 1.40 | **1.05 (1.01-1.09)** | 832 | 0.21 | 1.09 (0.99-1.20) |
| Q3 | 7835 | 2.00 | **1.09 (1.06-1.13)** | 5896 | 1.51 | **1.10 (1.06-1.14)** | 873 | 0.22 | **1.12 (1.02-1.23)** |
| Q4 | 10178 | 2.64 | **1.22 (1.18-1.26)** | 7524 | 1.95 | **1.20 (1.16-1.24)** | 1119 | 0.29 | **1.26 (1.15-1.38)** |
| *P* for trend | |  | < 0.001 |  |  | < 0.001 |  |  | < 0.001 |

Model 1(adjusted for age, sex, smoking, alcohol consumption, regular exercise and income)

**Table S3** Hazard ratios and 95% confidence intervals of all-cause dementia, Alzheimer’s disease and vascular dementia by quartiles of metabolic parameter variability measured as standard deviation

|  | **All-cause dementia** | | | **Alzheimer’s disease** | | | **Vascular dementia** | | |
| --- | --- | --- | --- | --- | --- | --- | --- | --- | --- |
|  | Events (n) | Incidence rate (per 1000  person-years) | HR (95% CI) | Events (n) | Incidence rate (per 1000  person-years) | HR (95% CI) | Events (n) | Incidence rate (per 1000  person-years) | HR (95% CI) |
| **Body mass index** | | | | | | | | | |
| Q1 | 6062 | 1.57 | 1 (ref) | 4556 | 1.18 | 1 (ref) | 661 | 0.17 | 1 (ref) |
| Q2 | 6533 | 1.66 | **1.08 (1.04-1.12)** | 4860 | 1.24 | **1.07 (1.02-1.11)** | 731 | 0.19 | 1.10 (0.99-1.22) |
| Q3 | 7752 | 1.98 | **1.15 (1.11-1.19)** | 5734 | 1.47 | **1.12 (1.08-1.17)** | 879 | 0.22 | **1.23 (1.11-1.36)** |
| Q4 | 12554 | 3.28 | **1.41 (1.36-1.45)** | 9336 | 2.44 | **1.36 (1.31-1.41)** | 1358 | 0.35 | **1.54 (1.40-1.69)** |
| *P* for trend | |  | < 0.001 |  |  | < 0.001 |  |  | < 0.001 |
| **Systolic blood pressure** | | | | | | | | | |
| Q1 | 6374 | 1.67 | 1 (ref) | 4707 | 1.23 | 1 (ref) | 720 | 0.19 | 1 (ref) |
| Q2 | 6955 | 1.77 | 1.02 (0.98-1.05) | 5177 | 1.32 | 1.02 (0.98-1.07) | 750 | 0.19 | 0.98 (0.89-1.09) |
| Q3 | 7204 | 1.83 | 1.03 (1.00-1.07) | 5350 | 1.36 | 1.03 (0.99-1.08) | 822 | 0.21 | 1.04 (0.94-1.15) |
| Q4 | 12368 | 3.21 | **1.16 (1.12-1.19)** | 9252 | 2.40 | **1.15 (1.11-1.19)** | 1337 | 0.35 | **1.19 (1.09-1.30)** |
| *P* for trend | |  | < 0.001 |  |  | < 0.001 |  |  | < 0.001 |
| **Glucose** | | | | | | | | | |
| Q1 | 7265 | 1.91 | 1 (ref) | 5378 | 1.42 | 1 (ref) | 801 | 0.21 | 1 (ref) |
| Q2 | 7518 | 1.93 | **1.05 (1.02-1.09)** | 5611 | 1.44 | **1.06 (1.02-1.10)** | 829 | 0.21 | 1.04 (0.94-1.15) |
| Q3 | 8076 | 2.05 | **1.07 (1.04-1.10)** | 5957 | 1.51 | **1.07 (1.03-1.11)** | 927 | 0.24 | **1.10 (1.00-1.21)** |
| Q4 | 10042 | 2.58 | **1.12 (1.09-1.16)** | 7540 | 1.93 | **1.14 (1.10-1.18)** | 1072 | 0.28 | **1.11 (1.01-1.22)** |
| *P* for trend | |  | < 0.001 |  |  | < 0.001 |  |  | 0.016 |
| **Total cholesterol** | | | | | | | | | |
| Q1 | 7533 | 1.95 | 1 (ref) | 5625 | 1.46 | 1 (ref) | 821 | 0.21 | 1 (ref) |
| Q2 | 7545 | 1.91 | **1.07 (1.04-1.10)** | 5614 | 1.42 | **1.07 (1.03-1.11)** | 837 | 0.21 | 1.07 (0.97-1.18) |
| Q3 | 7880 | 2.01 | **1.10 (1.06-1.13)** | 5855 | 1.50 | **1.09 (1.05-1.13)** | 920 | 0.23 | **1.16 (1.06-1.28)** |
| Q4 | 9943 | 2.60 | **1.22 (1.18-1.25)** | 7392 | 1.93 | **1.21 (1.16-1.25)** | 1051 | 0.27 | **1.20 (1.09-1.32)** |
| *P* for trend | |  | < 0.001 |  |  | < 0.001 |  |  | < 0.001 |

Adjusted for age, sex, smoking, alcohol consumption, regular exercise, income, glucose, systolic blood pressure, total cholesterol and body mass index (model 2)

**Table S4 Hazard ratios and 95% confidence intervals of all-cause dementia, Alzheimer’s disease and vascular dementia by quartiles of metabolic parameter variability measured as variability independent of the mean**

|  | **All-cause dementia** | | | **Alzheimer’s disease** | | | **Vascular dementia** | | |
| --- | --- | --- | --- | --- | --- | --- | --- | --- | --- |
|  | Events (n) | Incidence rate (per 1000  person-years) | HR (95% CI) | Events (n) | Incidence rate (per 1000  person-years) | HR (95% CI) | Events (n) | Incidence rate (per 1000  person-years) | HR (95% CI) |
| **Body mass index** | | | | | | | | | |
| Q1 | 5803 | 1.50 | 1 (ref) | 4327 | 1.12 | 1 (ref) | 653 | 0.17 | 1 (ref) |
| Q2 | 6240 | 1.59 | **1.07 (1.03-1.11)** | 4669 | 1.19 | **1.07 (1.03-1.11)** | 698 | 0.18 | 1.06 (0.96-1.18) |
| Q3 | 7445 | 1.90 | **1.13 (1.09-1.17)** | 5501 | 1.41 | **1.11 (1.07-1.15)** | 845 | 0.22 | **1.19 (1.07-1.31)** |
| Q4 | 13413 | 3.50 | **1.40 (1.36-1.45)** | 9989 | 2.61 | **1.36 (1.31-1.41)** | 1433 | 0.37 | **1.53 (1.39-1.68)** |
| *P* for trend | |  | < 0.001 |  |  | < 0.001 |  |  | < 0.001 |
| **Systolic blood pressure** | | | | | | | | | |
| Q1 | 6684 | 1.74 | 1 (ref) | 4937 | 1.28 | 1 (ref) | 752 | 0.20 | 1 (ref) |
| Q2 | 6870 | 1.76 | 1.03 (0.99-1.06) | 5106 | 1.31 | 1.03 (0.99-1.07) | 751 | 0.19 | 1.01 (0.91-1.11) |
| Q3 | 7385 | 1.88 | **1.03 (1.00-1.07)** | 5479 | 1.39 | 1.04 (1.00-1.08) | 861 | 0.22 | 1.08 (0.98-1.19) |
| Q4 | 11962 | 3.10 | **1.16 (1.13-1.20)** | 8964 | 2.32 | **1.16 (1.12-1.20)** | 1265 | 0.33 | **1.18 (1.08-1.29)** |
| *P* for trend | |  | < 0.001 |  |  | < 0.001 |  |  | < 0.001 |
| **Glucose** | | | | | | | | | |
| Q1 | 7476 | 1.97 | 1 (ref) | 5498 | 1.45 | 1 (ref) | 814 | 0.22 | 1 (ref) |
| Q2 | 7651 | 1.98 | **1.06 (1.02-1.09)** | 5698 | 1.47 | **1.07 (1.03-1.11)** | 859 | 0.22 | 1.07 (0.98-1.18) |
| Q3 | 8210 | 2.09 | **1.07 (1.04-1.10)** | 6123 | 1.56 | **1.08 (1.04-1.12)** | 935 | 0.24 | **1.12 (1.02-1.23)** |
| Q4 | 9564 | 2.41 | **1.11 (1.07-1.14)** | 7167 | 1.81 | **1.12 (1.08-1.16)** | 1021 | 0.26 | 1.10 (1.00-1.20) |
| *P* for trend | |  | < 0.001 |  |  | < 0.001 |  |  | 0.045 |
| **Total cholesterol** | | | | | | | | | |
| Q1 | 7504 | 1.95 | 1 (ref) | 5620 | 1.46 | 1 (ref) | 798 | 0.21 | 1 (ref) |
| Q2 | 7408 | 1.89 | **1.06 (1.03-1.09)** | 5469 | 1.39 | **1.05 (1.01-1.09)** | 846 | 0.22 | **1.12 (1.02-1.23)** |
| Q3 | 7815 | 2.00 | **1.09 (1.06-1.13)** | 5891 | 1.50 | **1.10 (1.06-1.14)** | 867 | 0.22 | **1.12 (1.02-1.24)** |
| Q4 | 10174 | 2.65 | **1.21 (1.18-1.25)** | 7506 | 1.95 | **1.19 (1.15-1.23)** | 1118 | 0.29 | **1.27 (1.16-1.40)** |
| *P* for trend | |  | < 0.001 |  |  | < 0.001 |  |  | < 0.001 |

Adjusted for age, sex, smoking, alcohol consumption, regular exercise, income, glucose, systolic blood pressure, total cholesterol and body mass index (model 2)

**Table S5** Hazard ratios and 95% confidence intervals of all-cause dementia, Alzheimer’s disease and vascular dementia by number of parameters with high variability (Covariates selected by backward stepwise elimination)

|  | **All-cause dementia** | **Alzheimer’s disease** | **Vascular dementia** |
| --- | --- | --- | --- |
| Number of high variability parameters |  |  |  |
| 0 | 1 (ref) | 1 (ref) | 1 (ref) |
| 1 | 1.22 (1.19-1.26) | 1.23 (1.19-1.27) | 1.19 (1.09-1.29) |
| 2 | 1.39 (1.35-1.43) | 1.37 (1.32-1.42) | 1.34 (1.22-1.48) |
| 3 | 1.54 (1.48-1.60) | 1.50 (1.43-1.57) | 1.57 (1.39-1.77) |
| 4 | 1.73 (1.60-1.88) | 1.65 (1.50-1.81) | 2.19 (1.74-2.75) |
| Age | 1.18 (1.18-1.18) | 1.18 (1.18-1.19) | 1.15 (1.14-1.15) |
| Sex | 1.45 (1.41-1.48) | 1.54 (1.50-1.58) | 1.16 (1.08-1.25) |
| Smoking | 1.15 (1.12-1.19) | 1.10 (1.06-1.15) | 1.53 (1.40-1.67) |
| Alcohol consumption | 1.11 (1.05-1.16) | 1.08 (1.02-1.15) | 0.89 (0.81-0.97) |
| Regular exercise | 0.90 (0.87-0.92) | 0.91 (0.88-0.94) |  |
| Body mass index | 0.98 (0.97-0.98) | 0.98 (0.97-0.98) |  |
| Glucose | 1.00 (1.00-1.00) | 1.00 (1.00-1.00) |  |
| Systolic blood pressure |  | 0.99 (0.99-1.00) | 1.01 (1.01-1.01) |
| Total cholesterol | 0.99 (0.99-1.00) | 0.99 (0.99-1.00) |  |

**Table S6** Hazard ratios and 95% confidence intervals of all-cause dementia, Alzheimer’s disease and vascular dementia by number of parameters with high variability measured as standard deviation

| n | Events (n) | Incidence rate (per 1000  person-years) | Model 1 | Model 2 |
| --- | --- | --- | --- | --- |
| **All-cause dementia** | | | | |
| 0 | 6810 | 1.28 | 1 (ref) | 1 (ref) |
| 1 | 12253 | 2.01 | **1.21 (1.17-1.25)** | **1.21 (1.18-1.25)** |
| 2 | 9499 | 3.00 | **1.37 (1.33-1.42)** | **1.38 (1.33-1.42)** |
| 3 | 3700 | 4.32 | **1.52 (1.46-1.58)** | **1.52 (1.46-1.58)** |
| 4 | 639 | 6.25 | **1.81 (1.66-1.96)** | **1.80 (1.66-1.96)** |
| *P* for trend | |  | < 0.001 | < 0.001 |
| **Alzheimer’s disease** | | | | |
| 0 | 4988 | 0.94 | 1 (ref) | 1 (ref) |
| 1 | 9161 | 1.50 | **1.22 (1.18-1.26)** | **1.22 (1.18-1.27)** |
| 2 | 7115 | 2.25 | **1.37 (1.32-1.42)** | **1.37 (1.32-1.43)** |
| 3 | 2759 | 3.22 | **1.49 (1.42-1.57)** | **1.49 (1.42-1.56)** |
| 4 | 463 | 4.53 | **1.72 (1.56-1.89)** | **1.72 (1.56-1.89)** |
| *P* for trend | |  | < 0.001 | < 0.001 |
| **Vascular dementia** | | | | |
| 0 | 824 | 0.16 | 1 (ref) | 1 (ref) |
| 1 | 1345 | 0.22 | **1.16 (1.06-1.26)** | **1.15 (1.06-1.26)** |
| 2 | 986 | 0.31 | **1.31 (1.19-1.43)** | **1.30 (1.18-1.43)** |
| 3 | 395 | 0.46 | **1.55 (1.37-1.75)** | **1.54 (1.36-1.74)** |
| 4 | 79 | 0.77 | **2.18 (1.73-2.75)** | **2.17 (1.72-2.74)** |
| *P* for trend | |  | < 0.001 | < 0.001 |

Model 1: adjusted for age, sex, smoking, alcohol consumption, regular exercise and income

Model 2: adjusted for model 1 plus glucose, systolic blood pressure, total cholesterol and body mass index

**Table S7** Hazard ratios and 95% confidence intervals of all-cause dementia, Alzheimer’s disease and vascular dementia by number of parameters with high variability measured as variability independent of the mean

| n | Events (n) | Incidence rate (per 1000  person-years) | Model 1 | Model 2 |
| --- | --- | --- | --- | --- |
| **All-cause dementia** | | | | |
| 0 | 6734 | 1.28 | 1 (ref) | 1 (ref) |
| 1 | 12241 | 2.00 | **1.22 (1.19-1.26)** | **1.22 (1.18-1.25)** |
| 2 | 9528 | 2.99 | **1.40 (1.36-1.44)** | **1.38 (1.34-1.43)** |
| 3 | 3776 | 4.39 | **1.56 (1.50-1.62)** | **1.53 (1.47-1.59)** |
| 4 | 622 | 6.15 | **1.73 (1.59-1.88)** | **1.69 (1.56-1.83)** |
| *P* for trend | |  | < 0.001 | < 0.001 |
| **Alzheimer’s disease** | | | | |
| 0 | 4970 | 0.95 | 1 (ref) | 1 (ref) |
| 1 | 9134 | 1.49 | **1.22 (1.18-1.26)** | **1.21 (1.17-1.26)** |
| 2 | 7111 | 2.23 | **1.38 (1.33-1.43)** | **1.36 (1.31-1.41)** |
| 3 | 2814 | 3.27 | **1.52 (1.45-1.59)** | **1.49 (1.42-1.56)** |
| 4 | 457 | 4.52 | **1.65 (1.50-1.82)** | **1.61 (1.46-1.77)** |
| *P* for trend | |  | < 0.001 | < 0.001 |
| **Vascular dementia** | | | | |
| 0 | 804 | 0.15 | 1 (ref) | 1 (ref) |
| 1 | 1360 | 0.22 | **1.20 (1.10-1.31)** | **1.20 (1.10-1.31)** |
| 2 | 996 | 0.31 | **1.36 (1.24-1.50)** | **1.36 (1.24-1.49)** |
| 3 | 391 | 0.45 | **1.57 (1.39-1.77)** | **1.56 (1.38-1.77)** |
| 4 | 78 | 0.77 | **2.16 (1.71-2.73)** | **2.15 (1.70-2.72)** |
| *P* for trend | |  | < 0.001 | < 0.001 |

Model 1: adjusted for age, sex, smoking, alcohol consumption, regular exercise and income

Model 2: adjusted for model 1 plus glucose, systolic blood pressure, total cholesterol and body mass index

**Table S8** Hazard ratios and 95% confidence intervals of all-cause dementia, Alzheimer’s disease and vascular dementia by number of parameters with high variability (Sensitivity analysis excluding subjects with the occurrence of end points within 3 years of follow-up)

|  | **All-cause dementia** | | | **Alzheimer’s disease** | | | **Vascular dementia** | | |
| --- | --- | --- | --- | --- | --- | --- | --- | --- | --- |
|  | Events (n) | Incidence rate (per 1000  person-years) | HR (95% CI) | Events (n) | Incidence rate (per 1000  person-years) | HR (95% CI) | Events (n) | Incidence rate (per 1000  person-years) | HR (95% CI) |
| **CV** | | | | | | | | | |
| 0 | 4013 | 1.74 | 1 (ref) | 2974 | 1.29 | 1 (ref) | 489 | 0.21 | 1 (ref) |
| 1 | 7144 | 2.67 | **1.21 (1.16-1.26)** | 5464 | 2.04 | **1.23 (1.18-1.29)** | 793 | 0.30 | **1.16 (1.04-1.30)** |
| 2 | 5421 | 3.91 | **1.34 (1.29-1.40)** | 4184 | 3.02 | **1.36 (1.30-1.42)** | 533 | 0.38 | **1.21 (1.07-1.37)** |
| 3 | 2123 | 5.69 | **1.48 (1.41-1.57)** | 1595 | 4.28 | **1.45 (1.36-1.54)** | 227 | 0.61 | **1.53 (1.30-1.79)** |
| 4 | 347 | 7.85 | **1.59 (1.43-1.78)** | 259 | 5.86 | **1.53 (1.35-1.74)** | 45 | 1.02 | **2.05 (1.51-2.78)** |
| *P* for trend | |  | < 0.001 |  |  | < 0.001 |  |  | < 0.001 |
| **SD** | | | | | | | | | |
| 0 | 4060 | 1.73 | 1 (ref) | 2990 | 1.28 | 1 (ref) | 500 | 0.21 | 1 (ref) |
| 1 | 7163 | 2.69 | **1.20 (1.15-1.25)** | 5488 | 2.06 | **1.23 (1.18-1.29)** | 774 | 0.29 | 1.11 (0.99-1.24) |
| 2 | 5416 | 3.95 | **1.34 (1.28-1.40)** | 4172 | 3.04 | **1.37 (1.30-1.43)** | 551 | 0.40 | **1.22 (1.08-1.38)** |
| 3 | 2062 | 5.60 | **1.46 (1.38-1.54)** | 1570 | 4.26 | **1.46 (1.37-1.55)** | 218 | 0.59 | **1.45 (1.23-1.70)** |
| 4 | 347 | 7.92 | **1.70 (1.52-1.89)** | 256 | 5.84 | **1.63 (1.44-1.86)** | 44 | 1.00 | **2.07 (1.52-2.82)** |
| *P* for trend | |  | < 0.001 |  |  | < 0.001 |  |  | < 0.001 |
| **VIM** | | | | | | | | | |
| 0 | 4008 | 1.74 | 1 (ref) | 2988 | 1.30 | 1 (ref) | 478 | 0.21 | 1 (ref) |
| 1 | 7142 | 2.67 | **1.20 (1.15-1.25)** | 5445 | 2.03 | **1.21 (1.15-1.26)** | 794 | 0.30 | **1.18 (1.06-1.33)** |
| 2 | 5424 | 3.89 | **1.33 (1.28-1.39)** | 4176 | 3.00 | **1.34 (1.28-1.40)** | 547 | 0.39 | **1.26 (1.11-1.43)** |
| 3 | 2140 | 5.70 | **1.48 (1.41-1.56)** | 1622 | 4.32 | **1.45 (1.36-1.54)** | 223 | 0.59 | **1.52 (1.29-1.78)** |
| 4 | 334 | 7.58 | **1.56 (1.39-1.74)** | 245 | 5.56 | **1.46 (1.28-1.67)** | 45 | 1.02 | **2.12 (1.56-2.88)** |
| *P* for trend | |  | < 0.001 |  |  | < 0.001 |  |  | < 0.001 |

Adjusted for age, sex, smoking, alcohol consumption, regular exercise, income, glucose, systolic blood pressure, total cholesterol and body mass index (model 2)

**Table S9** Hazard ratios and 95% confidence intervals of all-cause dementia, Alzheimer’s disease and vascular dementia by number of parameters with high variability (Sensitivity analysis excluding diseases known as a risk factor of dementia)

|  | **All-cause dementia** | | | **Alzheimer’s disease** | | | **Vascular dementia** | | |
| --- | --- | --- | --- | --- | --- | --- | --- | --- | --- |
|  | Events (n) | Incidence rate (per 1000  person-years) | HR (95% CI) | Events (n) | Incidence rate (per 1000  person-years) | HR (95% CI) | Events (n) | Incidence rate (per 1000  person-years) | HR (95% CI) |
| **CV** | | | | | | | | | |
| 0 | 5474 | 1.12 | 1 (ref) | 4015 | 0.82 | 1 (ref) | 663 | 0.14 | 1 (ref) |
| 1 | 9766 | 1.73 | **1.22 (1.18-1.26)** | 7348 | 1.30 | **1.23 (1.19-1.28)** | 1101 | 0.19 | **1.17 (1.06-1.29)** |
| 2 | 7387 | 2.55 | **1.35 (1.31-1.40)** | 5550 | 1.92 | **1.35 (1.29-1.40)** | 770 | 0.27 | **1.30 (1.17-1.44)** |
| 3 | 2840 | 3.68 | **1.47 (1.40-1.54)** | 2111 | 2.74 | **1.43 (1.35-1.51)** | 307 | 0.40 | **1.50 (1.31-1.73)** |
| 4 | 501 | 5.53 | **1.72 (1.56-1.88)** | 360 | 3.97 | **1.60 (1.44-1.79)** | 63 | 0.70 | **2.21 (1.71-2.86)** |
| *P* for trend | |  | < 0.001 |  |  | < 0.001 |  |  | < 0.001 |
| **SD** | | | | | | | | | |
| 0 | 5535 | 1.12 | 1 (ref) | 4059 | 0.82 | 1 (ref) | 679 | 0.14 | 1 (ref) |
| 1 | 9786 | 1.74 | **1.20 (1.16-1.24)** | 7347 | 1.31 | **1.21 (1.17-1.26)** | 1087 | 0.19 | **1.14 (1.04-1.26)** |
| 2 | 7355 | 2.55 | **1.33 (1.29-1.38)** | 5546 | 1.92 | **1.34 (1.28-1.39)** | 768 | 0.27 | **1.25 (1.13-1.39)** |
| 3 | 2798 | 3.63 | **1.46 (1.39-1.53)** | 2074 | 2.69 | **1.42 (1.35-1.50)** | 310 | 0.40 | **1.52 (1.32-1.74)** |
| 4 | 494 | 5.41 | **1.77 (1.62-1.95)** | 358 | 3.92 | **1.68 (1.51-1.88)** | 60 | 0.66 | **2.07 (1.59-2.70)** |
| *P* for trend | |  | < 0.001 |  |  | < 0.001 |  |  | < 0.001 |
| **VIM** | | | | | | | | | |
| 0 | 5463 | 1.12 | 1 (ref) | 4032 | 0.82 | 1 (ref) | 675 | 0.14 | 1 (ref) |
| 1 | 9810 | 1.74 | **1.22 (1.18-1.26)** | 7353 | 1.30 | **1.22 (1.17-1.27)** | 1089 | 0.19 | **1.20 (1.09-1.32)** |
| 2 | 7376 | 2.54 | **1.35 (1.30-1.39)** | 5544 | 1.91 | **1.33 (1.28-1.38)** | 774 | 0.27 | **1.31 (1.18-1.46)** |
| 3 | 2843 | 3.68 | **1.47 (1.40-1.54)** | 2113 | 2.73 | **1.41 (1.34-1.49)** | 301 | 0.39 | **1.56 (1.36-1.79)** |
| 4 | 476 | 5.30 | **1.67 (1.52-1.83)** | 342 | 3.81 | **1.55 (1.39-1.73)** | 65 | 0.72 | **2.23 (1.72-2.90)** |
| *P* for trend | |  | < 0.001 |  |  | < 0.001 |  |  | < 0.001 |

Adjusted for age, sex, smoking, alcohol consumption, regular exercise, income, glucose, systolic blood pressure, total cholesterol and body mass index (model 2)

**Table S10** Hazard ratios and 95% confidence intervals of all-cause dementia, Alzheimer’s disease and vascular dementia by number of parameters with high variability measured as coefficient of variation (Further adjusted for diseases known as a risk factor of dementia)

| n | Events (n) | Incidence rate (per 1000  person-years) | HR (95% CI) | | |  |
| --- | --- | --- | --- | --- | --- | --- |
| **All-cause dementia** | | | |  |  | |
| 0 | 6744 | 1.28 | 1 (ref) | | |  |
| 1 | 12195 | 1.99 | **1.22 (1.18-1.25)** | | |  |
| 2 | 9528 | 3.00 | **1.37 (1.33-1.42)** | | |  |
| 3 | 3783 | 4.40 | **1.51 (1.45-1.57)** | | |  |
| 4 | 651 | 6.38 | **1.69 (1.56-1.83)** | | |  |
| *P* for trend | |  |  | | |  |
| **Alzheimer’s disease** | | | |  | | |
| 0 | 4952 | 0.94 | 1 (ref) | | |  |
| 1 | 9136 | 1.49 | **1.22 (1.18-1.27)** | | |  |
| 2 | 7106 | 2.24 | **1.36 (1.31-1.41)** | | |  |
| 3 | 2818 | 3.28 | **1.48 (1.41-1.55)** | | |  |
| 4 | 474 | 4.64 | **1.61 (1.46-1.77)** | | |  |
| *P* for trend | |  | < 0.001 | | |  |
| **Vascular dementia** | | | |  | | |
| 0 | 815 | 0.16 | 1 (ref) | | |  |
| 1 | 1350 | 0.22 | **1.18 (1.08-1.28)** | | |  |
| 2 | 990 | 0.31 | **1.32 (1.21-1.45)** | | |  |
| 3 | 393 | 0.46 | **1.53 (1.35-1.73)** | | |  |
| 4 | 81 | 0.79 | **2.11 (1.68-2.66)** | | |  |
| *P* for trend | |  | < 0.001 | | |  |

Adjusted for age, sex, smoking, alcohol consumption, regular exercise, income, glucose, systolic blood pressure, total cholesterol, body mass index, ischemic heart disease, stroke, depression, head injury, Parkinson’s disease and HIV infection

**Table S11** Hazard ratios and 95% confidence intervals of all-cause dementia, Alzheimer’s disease and vascular dementia by number of parameters with high variability (High variability defined as greater than 1 SD)

|  | **All-cause dementia** | | | **Alzheimer’s disease** | | | **Vascular dementia** | | |
| --- | --- | --- | --- | --- | --- | --- | --- | --- | --- |
|  | Events (n) | Incidence rate (per 1000  person-years) | HR (95% CI) | Events (n) | Incidence rate (per 1000  person-years) | HR (95% CI) | Events (n) | Incidence rate (per 1000  person-years) | HR (95% CI) |
| **CV** | | | | | | | | | |
| 0 | 13271 | 1.48 | 1 (ref) | 9813 | 1.09 | 1 (ref) | 1536 | 0.17 | 1 (ref) |
| 1 | 12724 | 2.53 | **1.22 (1.19-1.25)** | 9561 | 1.90 | **1.21 (1.18-1.25)** | 1397 | 0.28 | **1.24 (1.15-1.34)** |
| 2 | 5461 | 4.12 | **1.36 (1.31-1.40)** | 4044 | 3.05 | **1.31 (1.27-1.36)** | 539 | 0.41 | **1.33 (1.21-1.47)** |
| 3 | 1301 | 6.68 | **1.55 (1.46-1.64)** | 964 | 4.95 | **1.48 (1.39-1.59)** | 134 | 0.69 | **1.69 (1.41-2.02)** |
| 4 | 144 | 10.90 | **1.92 (1.63-2.27)** | 104 | 7.87 | **1.79 (1.47-2.17)** | 23 | 1.74 | **3.32 (2.19-5.01)** |
| *P* for trend | |  | < 0.001 |  |  | < 0.001 |  |  | < 0.001 |
| **SD** | | | | | | | | | |
| 0 | 14067 | 1.50 | 1 (ref) | 10437 | 1.12 | 1 (ref) | 1609 | 0.17 | 1 (ref) |
| 1 | 12575 | 2.61 | **1.20 (1.17-1.23)** | 9414 | 1.95 | **1.19 (1.16-1.23)** | 1377 | 0.29 | **1.23 (1.14-1.32)** |
| 2 | 5063 | 4.24 | **1.38 (1.33-1.42)** | 3752 | 3.15 | **1.33 (1.28-1.39)** | 499 | 0.42 | **1.35 (1.21-1.49)** |
| 3 | 1096 | 6.67 | **1.59 (1.49-1.69)** | 808 | 4.92 | **1.51 (1.41-1.63)** | 130 | 0.79 | **1.97 (1.64-2.36)** |
| 4 | 100 | 9.63 | **1.89 (1.56-2.31)** | 75 | 7.22 | **1.83 (1.46-2.30)** | 14 | 1.35 | **2.82 (1.66-4.77)** |
| *P* for trend | |  | < 0.001 |  |  | < 0.001 |  |  | < 0.001 |
| **VIM** | | | | | | | | | |
| 0 | 12948 | 1.48 | 1 (ref) | 9584 | 1.09 | 1 (ref) | 1497 | 0.17 | 1 (ref) |
| 1 | 12821 | 2.50 | **1.21 (1.18-1.24)** | 9610 | 1.87 | **1.21 (1.17-1.24)** | 1400 | 0.27 | **1.23 (1.14-1.32)** |
| 2 | 5650 | 4.01 | **1.36 (1.31-1.40)** | 4188 | 2.98 | **1.31 (1.27-1.36)** | 573 | 0.41 | **1.36 (1.24-1.50)** |
| 3 | 1327 | 6.28 | **1.51 (1.42-1.60)** | 991 | 4.69 | **1.46 (1.36-1.56)** | 138 | 0.65 | **1.64 (1.37-1.95)** |
| 4 | 155 | 10.66 | **1.81 (1.55-2.13)** | 113 | 7.77 | **1.69 (1.41-2.04)** | 21 | 1.44 | **2.67 (1.74-4.12)** |
| *P* for trend | |  | < 0.001 |  |  | < 0.001 |  |  | < 0.001 |

Adjusted for age, sex, smoking, alcohol consumption, regular exercise, income, glucose, systolic blood pressure, total cholesterol and body mass index (model 2)

**Table S12** Hazard ratios and 95% confidence intervals of all-cause dementia, Alzheimer’s disease and vascular dementia by weighted variability score

| Group* | Events (n) | Incidence rate (per 1000  person-years) | Model 1 | Model 2 |
| --- | --- | --- | --- | --- |
| **All-cause dementia** | | | | |
| Q1 | 6744 | 1.28 | 1 (ref) | 1 (ref) |
| Q2 | 2506 | 1.57 | **1.13 (1.08-1.19)** | **1.12 (1.07-1.17)** |
| Q3 | 5782 | 1.89 | **1.18 (1.14-1.22)** | **1.18 (1.13-1.22)** |
| Q4 | 6413 | 2.50 | **1.35 (1.30-1.40)** | **1.33 (1.29-1.38)** |
| Q5 | 11456 | 3.75 | **1.52 (1.47-1.57)** | **1.49 (1.45-1.54)** |
| *P* for trend | |  | < 0.001 | < 0.001 |
| **Alzheimer’s disease** | | | | |
| Q1 | 4952 | 0.94 | 1 (ref) | 1 (ref) |
| Q2 | 1899 | 1.19 | **1.17 (1.11-1.23)** | **1.15 (1.09-1.21)** |
| Q3 | 4318 | 1.41 | **1.19 (1.14-1.24)** | **1.18 (1.13-1.23)** |
| Q4 | 4803 | 1.87 | **1.35 (1.30-1.40)** | **1.33 (1.28-1.38)** |
| Q5 | 8514 | 2.79 | **1.49 (1.44-1.55)** | **1.46 (1.41-1.51)** |
| *P* for trend | |  | < 0.001 | < 0.001 |
| **Vascular dementia** | | | | |
| Q1 | 815 | 0.16 | 1 (ref) | 1 (ref) |
| Q2 | 287 | 0.18 | 1.07 (0.93-1.22) | 1.06 (0.93-1.22) |
| Q3 | 623 | 0.20 | 1.10 (0.99-1.22) | 1.10 (0.99-1.22) |
| Q4 | 704 | 0.27 | **1.33 (1.20-1.47)** | **1.32 (1.20-1.47)** |
| Q5 | 1200 | 0.39 | **1.50 (1.37-1.64)** | **1.50 (1.37-1.64)** |
| *P* for trend | |  | < 0.001 | < 0.001 |

Model 1: adjusted for age, sex, smoking, alcohol consumption, regular exercise and income

Model 2: adjusted for model 1 plus glucose, systolic blood pressure, total cholesterol and body mass index

***** Weighted variability score, corrected for the strength of the association of each metabolic parameter with the risk of dementia. For example, if the HR for highest quartile is 1.3, we gave 1.3 point instead of 1 point. After adding the points from four parameters, subjects were classified into quintile groups.

**Table S13** Hazard ratios and 95% confidence intervals of all-cause dementia, Alzheimer’s disease and vascular dementia by number of parameters with high variability measured as coefficient of variation (Sensitivity analysis censoring cases with incident diabetes mellitus, hypertension, or dyslipidemia during the follow-up period)

| n | Events (n) | Incidence rate (per 1000  person-years) | Model 1 | Model 2 |
| --- | --- | --- | --- | --- |
| **All-cause dementia** | | | | |
| 0 | 4824 | 0.92 | 1 (ref) | 1 (ref) |
| 1 | 8677 | 1.42 | **1.22 (1.18-1.27)** | **1.22 (1.18-1.25)** |
| 2 | 6852 | 2.16 | **1.41 (1.36-1.47)** | **1.38 (1.33-1.42)** |
| 3 | 2780 | 3.24 | **1.61 (1.54-1.69)** | **1.50 (1.43-1.56)** |
| 4 | 490 | 4.81 | **1.87 (1.70-2.05)** | **1.66 (1.53-1.81)** |
| *P* for trend | |  | < 0.001 | < 0.001 |
| **Alzheimer’s disease** | | | | |
| 0 | 3660 | 0.70 | 1 (ref) | 1 (ref) |
| 1 | 6665 | 1.09 | **1.23 (1.18-1.28)** | **1.22 (1.18-1.27)** |
| 2 | 5188 | 1.63 | **1.38 (1.32-1.44)** | **1.36 (1.31-1.41)** |
| 3 | 2137 | 2.49 | **1.59 (1.50-1.67)** | **1.46 (1.39-1.53)** |
| 4 | 370 | 3.63 | **1.80 (1.62-2.01)** | **1.58 (1.43-1.74)** |
| *P* for trend | |  | < 0.001 | < 0.001 |
| **Vascular dementia** | | | | |
| 0 | 490 | 0.09 | 1 (ref) | 1 (ref) |
| 1 | 785 | 0.13 | **1.14 (1.02-1.28)** | **1.18 (1.08-1.29)** |
| 2 | 612 | 0.19 | **1.37 (1.21-1.54)** | **1.33 (1.21-1.46)** |
| 3 | 235 | 0.27 | **1.53 (1.31-1.80)** | **1.52 (1.35-1.72)** |
| 4 | 46 | 0.45 | **2.03 (1.50-2.75)** | **2.09 (1.66-2.64)** |
| *P* for trend | |  | < 0.001 | < 0.001 |

Model 1: adjusted for age, sex, smoking, alcohol consumption, regular exercise and income

Model 2: adjusted for model 1 plus glucose, systolic blood pressure, total cholesterol and body mass index

**Table S14** Hazard ratios and 95% confidence intervals of all-cause dementia, Alzheimer’s disease and vascular dementia by number of parameters with high variability measured as coefficient of variation (Total population, Subjects with metabolic disease at baseline)

|  | Total population*  (n = 6,677,139) | | | | | Subjects with metabolic disease at baseline  (n = 3,746,323) | | |
| --- | --- | --- | --- | --- | --- | --- | --- | --- |
| n | Events (n) | Incidence rate (per 1000  person-years) | | HR (95% CI) | | Events (n) | Incidence rate (per 1000  person-years) | HR (95% CI) |
| **All-cause dementia** | | |  | |  | | | |
| 0 | 38016 | 3.18 | | 1 (ref) | | 31272 | 4.67 | 1 (ref) |
| 1 | 65446 | 4.76 | | **1.19 (1.17-1.20)** | | 53251 | 6.98 | **1.19 (1.17-1.21)** |
| 2 | 49207 | 6.91 | | **1.37 (1.35-1.39)** | | 39679 | 10.06 | **1.39 (1.37-1.41)** |
| 3 | 18707 | 9.62 | | **1.54 (1.51-1.57)** | | 14924 | 13.75 | **1.59 (1.56-1.62)** |
| 4 | 3020 | 12.82 | | **1.74 (1.67-1.80)** | | 2369 | 17.74 | **1.81 (1.73-1.88)** |
| *P* for trend | |  | | < 0.001 | |  |  | < 0.001 |
| **Alzheimer’s disease** | | | | |  | | | |
| 0 | 27543 | 2.30 | | 1 (ref) | | 22591 | 3.38 | 1 (ref) |
| 1 | 47991 | 3.49 | | **1.19 (1.17-1.21)** | | 38855 | 5.09 | **1.19 (1.17-1.21)** |
| 2 | 36059 | 5.06 | | **1.36 (1.34-1.39)** | | 28953 | 7.34 | **1.39 (1.36-1.41)** |
| 3 | 13663 | 7.03 | | **1.52 (1.49-1.56)** | | 10845 | 9.99 | **1.57 (1.54-1.61)** |
| 4 | 2190 | 9.29 | | **1.70 (1.63-1.78)** | | 1716 | 12.85 | **1.78 (1.69-1.87)** |
| *P* for trend | |  | | < 0.001 | |  |  | < 0.001 |
| **Vascular dementia** | | |  | |  | | | |
| 0 | 5003 | 0.42 | | 1 (ref) | | 4188 | 0.63 | 1 (ref) |
| 1 | 8131 | 0.59 | | **1.16 (1.12-1.20)** | | 6781 | 0.89 | **1.17 (1.13-1.22)** |
| 2 | 6026 | 0.85 | | **1.35 (1.30-1.40)** | | 5036 | 1.28 | **1.40 (1.34-1.46)** |
| 3 | 2297 | 1.18 | | **1.56 (1.48-1.64)** | | 1904 | 1.75 | **1.64 (1.55-1.74)** |
| 4 | 371 | 1.57 | | **1.77 (1.59-1.97)** | | 290 | 2.17 | **1.81 (1.60-2.04)** |
| *P* for trend | |  | | < 0.001 | |  |  | < 0.001 |

Adjusted for age, sex, smoking, alcohol consumption, regular exercise and income, glucose, systolic blood pressure, total cholesterol and body mass index

*Subjects with and without metabolic diseases (diabetes mellitus, hypertension, or dyslipidemia) at baseline

**Figure S1** Cumulative incidence of all-cause dementia, Alzheimer’s disease, and vascular dementia according to the number of metabolic parameters with high variability defined as the highest quartile of standard deviation


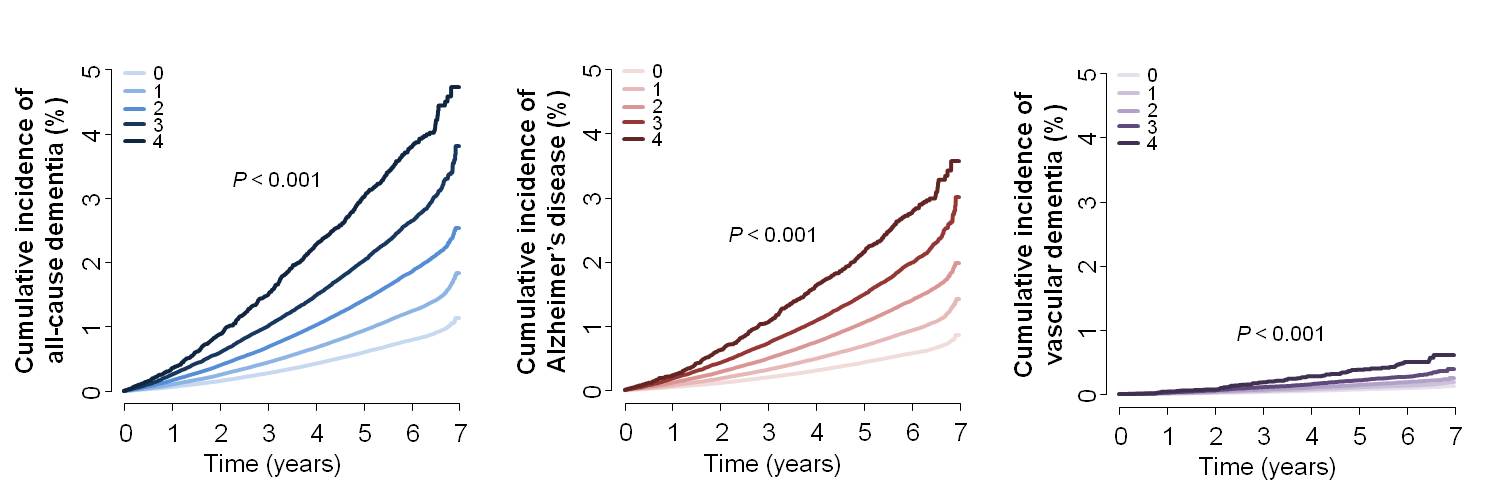


**Figure S2** Cumulative incidence of all-cause dementia, Alzheimer’s disease, and vascular dementia according to the number of metabolic parameters with high variability defined as the highest quartile of variability independent of the mean


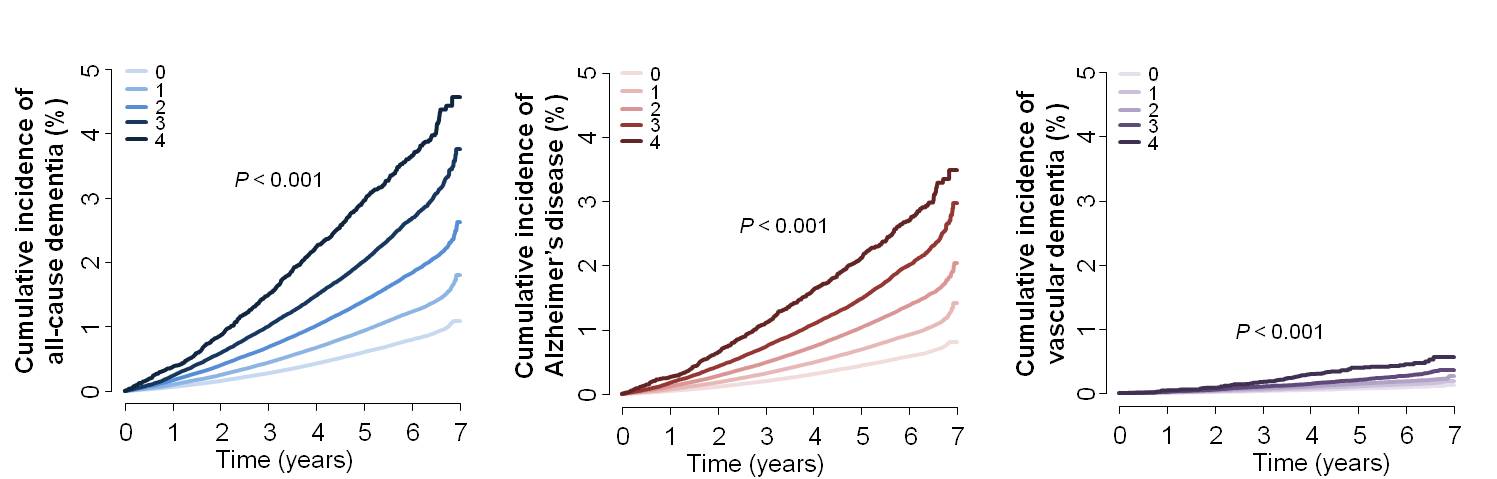


**Figure S3** Incidence rate, hazard ratios and 95% confidence intervals of all-cause dementia, Alzheimer’s disease, and vascular dementia according to the variability score (standard deviation)


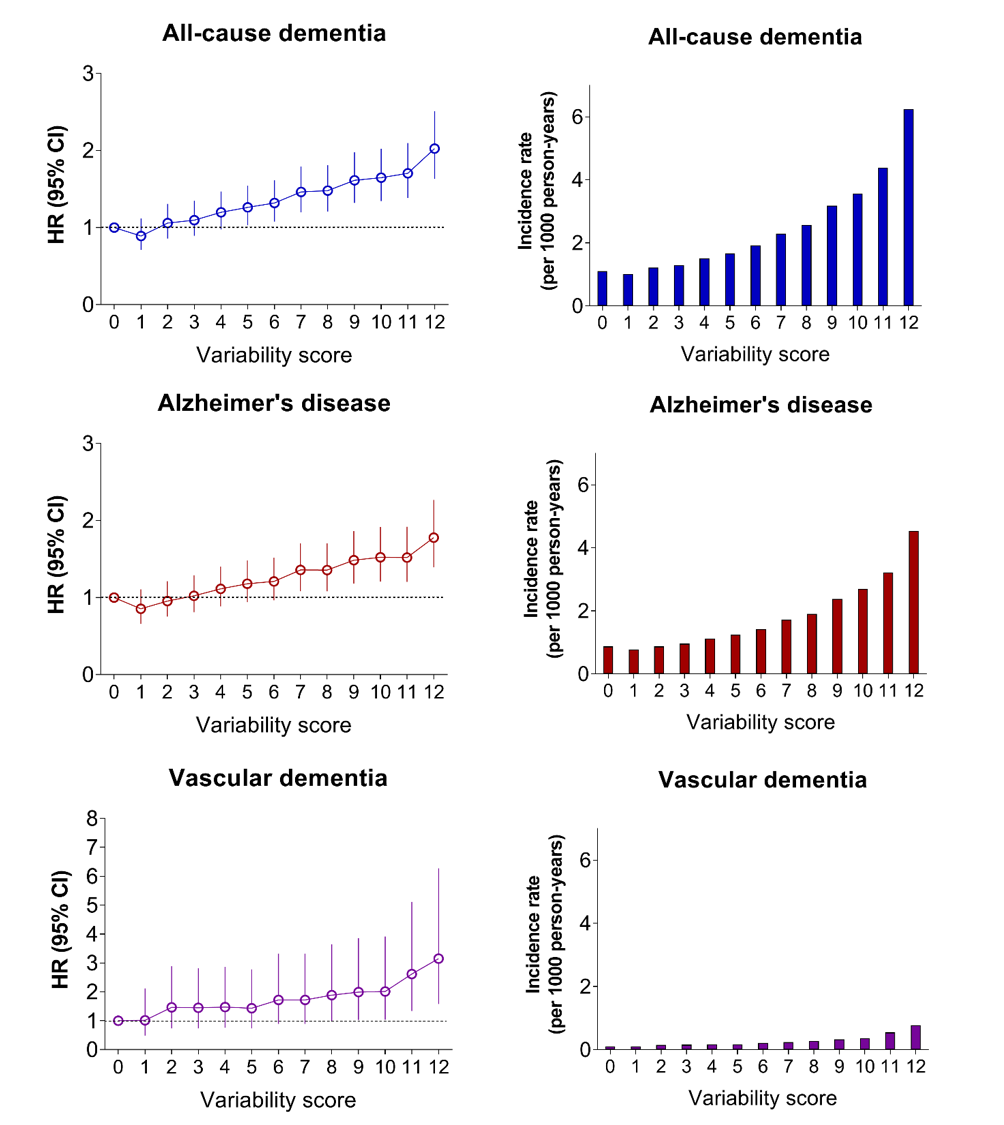


0 points were assigned for Q1 (lowest quartile of variability), 1 point for Q2, 2 points for Q3 and 3 points for Q4 (highest quartile of variability) for each of BP, glucose, cholesterol and body mass index variability measured as standard deviation. Total score ranged from 0 to 12.

Adjusted for age, sex, smoking, alcohol consumption, regular exercise, income, glucose, systolic blood pressure, total cholesterol and body mass index

**Figure S4** Incidence rate, hazard ratios and 95% confidence intervals of all-cause dementia, Alzheimer’s disease, and vascular dementia according to the variability score (variability independent of the mean)


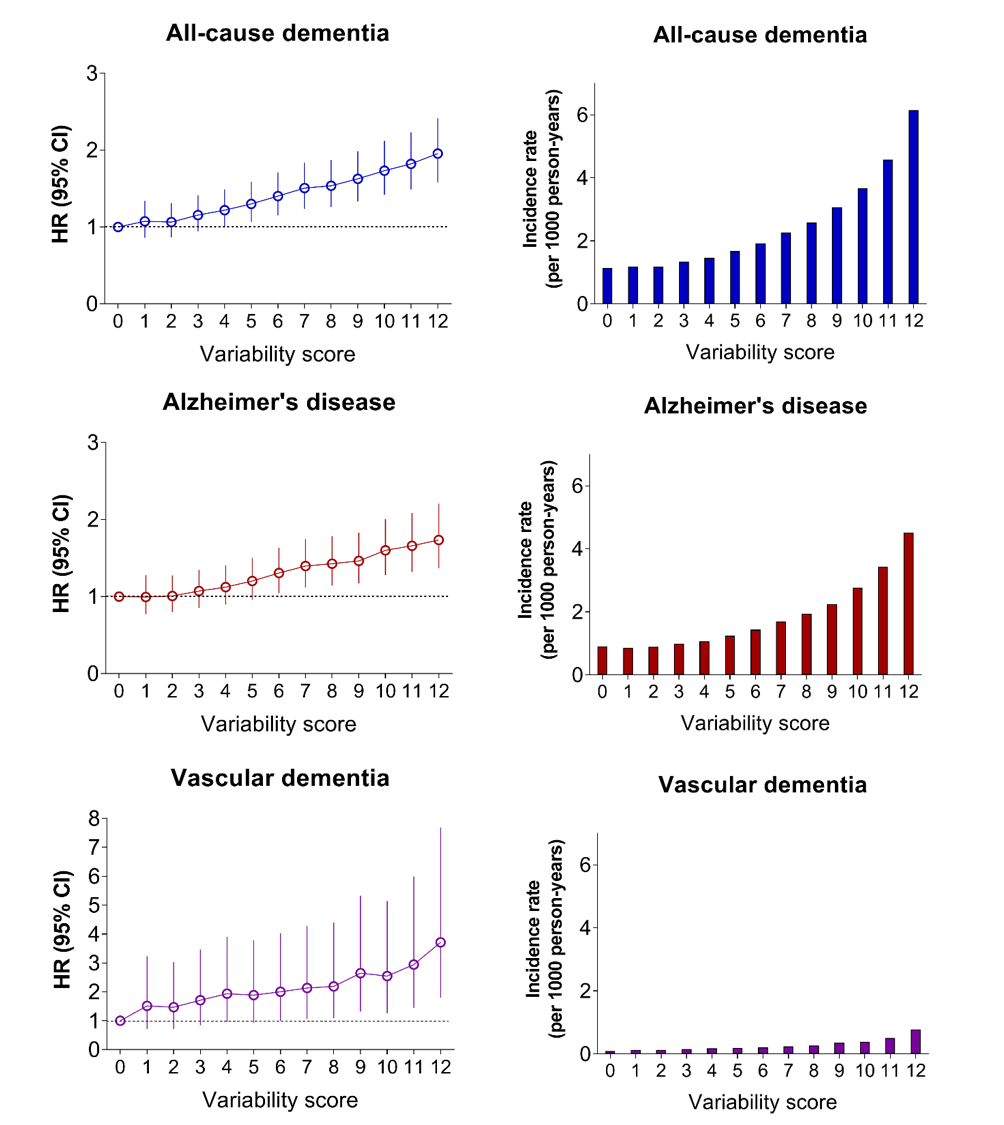


0 points were assigned for Q1 (lowest quartile of variability), 1 point for Q2, 2 points for Q3 and 3 points for Q4 (highest quartile of variability) for each of BP, glucose, cholesterol and body mass index variability measured as variability independent of the mean. Total score ranged from 0 to 12.

Adjusted for age, sex, smoking, alcohol consumption, regular exercise, income, glucose, systolic blood pressure, total cholesterol and body mass index

**Figure S5** Hazard ratios and 95% confidence intervals of all-cause dementia (A), Alzheimer’s disease (B), and vascular dementia (C) by the number of metabolic parameters with high variability defined as the highest quartile of standard deviation. Subgroup analyses according to age, sex and presence or absence of obesity

**
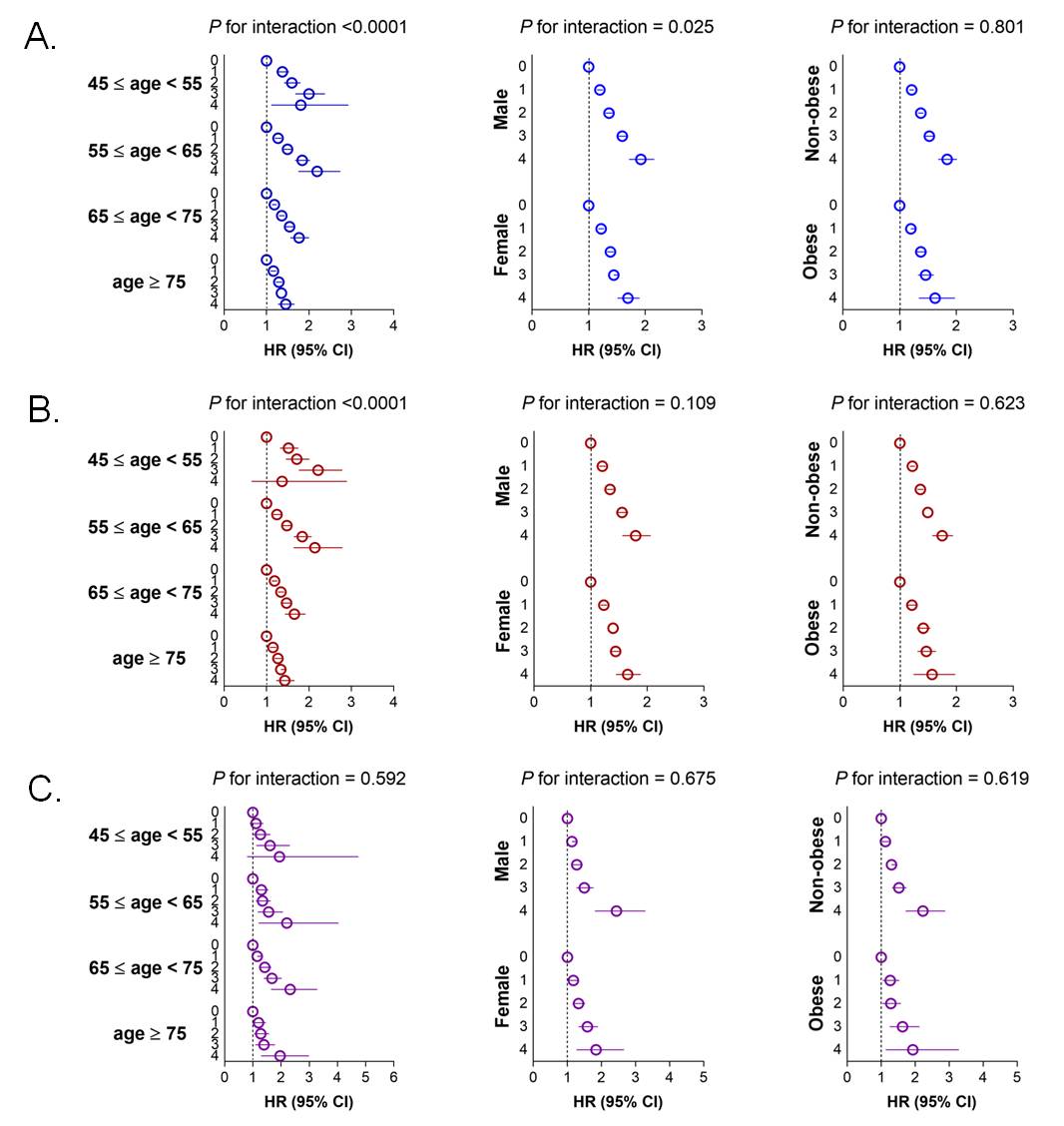
**

Adjusted for age, sex, smoking, alcohol consumption, regular exercise, income, glucose, systolic blood pressure, total cholesterol and body mass index

**Figure S6** Hazard ratios and 95% confidence intervals of all-cause dementia (A), Alzheimer’s disease (B), and vascular dementia (C) by the number of metabolic parameters with high variability defined as the highest quartile of variability independent of the mean. Subgroup analyses according to age, sex and presence or absence of obesity


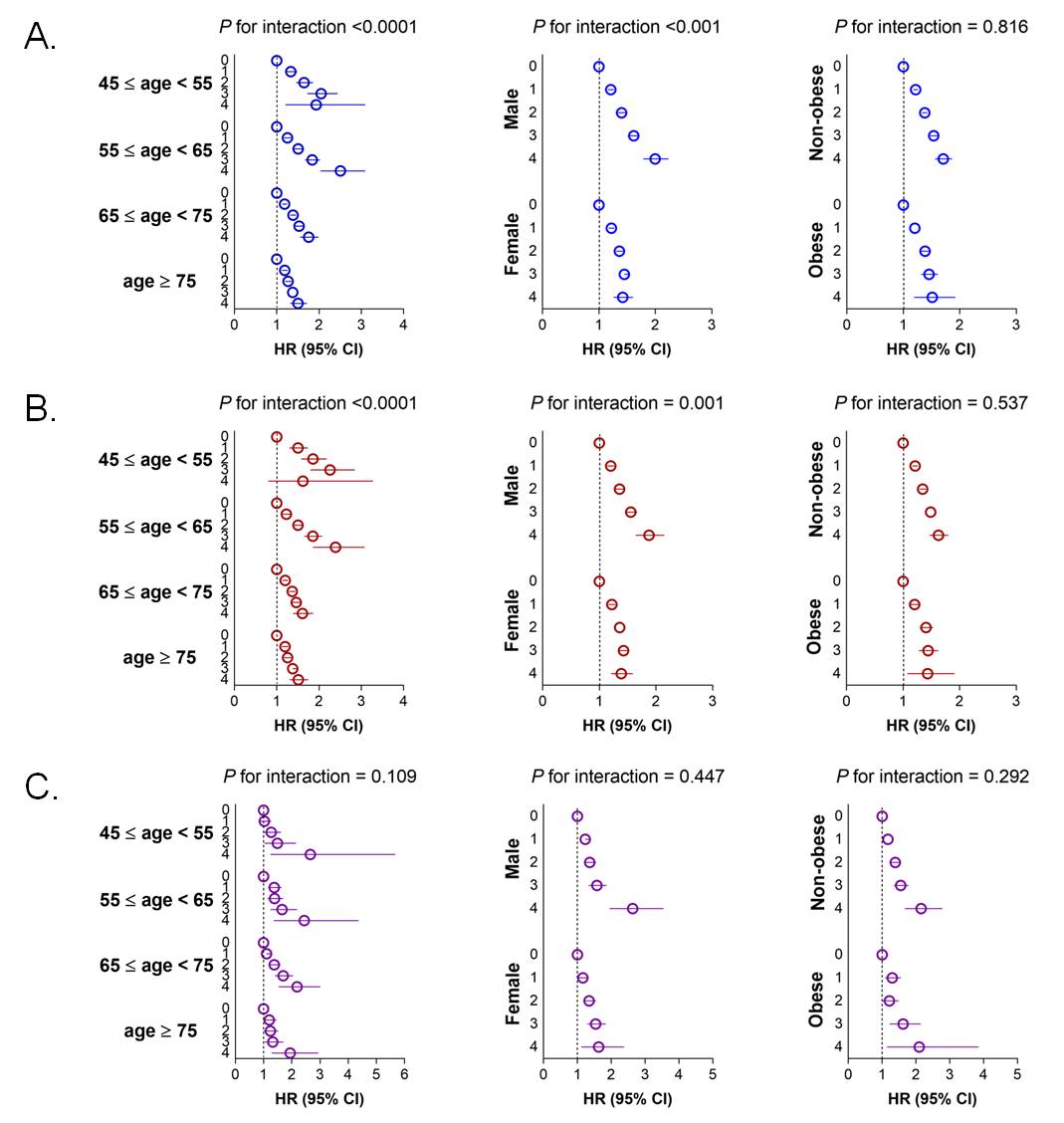


Adjusted for age, sex, smoking, alcohol consumption, regular exercise, income, glucose, systolic blood pressure, total cholesterol and body mass index
